# *Mycobacterium* *tuberculosis* Protein PE6 (Rv0335c), a Novel TLR4 Agonist, Evokes an Inflammatory Response and Modulates the Cell Death Pathways in Macrophages to Enhance Intracellular Survival

**DOI:** 10.3389/fimmu.2021.696491

**Published:** 2021-07-12

**Authors:** Neha Sharma, Mohd Shariq, Neha Quadir, Jasdeep Singh, Javaid A. Sheikh, Seyed E. Hasnain, Nasreen Z. Ehtesham

**Affiliations:** ^1^ Indian Council of Medical Research-National Institute of Pathology, New Delhi, India; ^2^ Jamia Hamdard Institute of Molecular Medicine, Jamia Hamdard, New Delhi, India; ^3^ Department of Biotechnology, School of Chemical and Life Science, Jamia Hamdard, New Delhi, India; ^4^ Department of Biochemical Engineering and Biotechnology, Indian Institute of Technology-Delhi, New Delhi, India; ^5^ Department of Life Science, School of Basic Science and Research, Sharda University, Greater Noida, India

**Keywords:** autophagy, apoptosis, proinflammatory cytokines, TLR4, iron acquisition

## Abstract

*Mycobacterium tuberculosis* (*M. tb*) is an intracellular pathogen that exploits moonlighting functions of its proteins to interfere with host cell functions. PE/PPE proteins utilize host inflammatory signaling and cell death pathways to promote pathogenesis. We report that *M. tb* PE6 protein (Rv0335c) is a secretory protein effector that interacts with innate immune toll-like receptor TLR4 on the macrophage cell surface and promotes activation of the canonical NFĸB signaling pathway to stimulate secretion of proinflammatory cytokines TNF-α, IL-12, and IL-6. Using mouse macrophage TLRs knockout cell lines, we demonstrate that PE6 induced secretion of proinflammatory cytokines dependent on TLR4 and adaptor Myd88. PE6 possesses nuclear and mitochondrial targeting sequences and displayed time-dependent differential localization into nucleus/nucleolus and mitochondria, and exhibited strong Nucleolin activation. PE6 strongly induces apoptosis *via* increased production of pro-apoptotic molecules Bax, Cytochrome C, and pcMyc. Mechanistic details revealed that PE6 activates Caspases 3 and 9 and induces endoplasmic reticulum-associated unfolded protein response pathways to induce apoptosis through increased production of ATF6, Chop, BIP, eIF2α, IRE1α, and Calnexin. Despite being a potent inducer of apoptosis, PE6 suppresses innate immune defense strategy autophagy by inducing inhibitory phosphorylation of autophagy initiating kinase ULK1. Inversely, PE6 induces activatory phosphorylation of autophagy master regulator MtorC1, which is reflected by lower conversion of autophagy markers LC3BI to LC3BII and increased accumulation of autophagy substrate p62 which is also dependent on innate immune receptor TLR4. The use of pharmacological agents, rapamycin and bafilomycin A1, confirms the inhibitory effect of PE6 on autophagy, evidenced by the reduced conversion of LC3BI to LC3BII and increased accumulation of p62 in the presence of rapamycin and bafilomycin A1. We also observed that PE6 binds DNA, which could have significant implications in virulence. Furthermore, our analyses reveal that PE6 efficiently binds iron to likely aid in intracellular survival. Recombinant *Mycobacterium smegmatis* (*M. smegmatis*) containing *pe6* displayed robust growth in iron chelated media compared to vector alone transformed cells, which suggests a role of PE6 in iron acquisition. These findings unravel novel mechanisms exploited by PE6 protein to subdue host immunity, thereby providing insights relevant to a better understanding of host–pathogen interaction during *M. tb* infection.

## Introduction

*M. tb* is the etiological agent of tuberculosis (TB) that remains the world’s most deadly infectious disease with the notorious distinction of being among the top ten causes of death worldwide. TB is a global pandemic, with the World Health Organization reporting 10 million infections and 1.4 million deaths in 2019 worldwide, with 21% (0.46 million) cases being drug-resistant TB (1). The co-occurrence of TB with the Human Immunodeficiency Virus and the emergence of multidrug-resistant, extensive-drug-resistant, and totally-drug-resistant strains are serious concerns and need urgent attention to curb this deadly menace (2). Recently, new vaccine development with novel modifications in the century-old bacille Calmette–Guerin (BCG) vaccine appears to increase its efficacy (3). Therefore, identification and characterization of virulence determinants of *M. tb* that play critical roles in immune responses and host–pathogen interaction are urgently warranted to get better insights to combat this deadly menace (4).

The sequencing of the *M. tb* genome in 1998 by Cole et al. identified an exciting family of genes, the Proline–Glutamic acid/Proline–Proline–Glutamic acid (PE/PPE) gene, family. These genes account for approximately 10% of the whole *M. tb* genome and are present preferentially in the pathogenic, slow-growing strains. Their name is derived from the conserved N-terminal amino acid sequence of Proline–Glutamic acid/Proline–Proline–Glutamic acid (PE/PPE) residues (5). Due to their exclusive presence in the virulent mycobacterial species, there has been a growing interest in understanding their role and action mechanism during *M. tb* infection. These proteins have been implicated in providing antigenic variation, immune evasion, virulence, and modulating the host cell death pathways (6, 7), contributing to the pathogen’s survival (8). Earlier reports suggested that PE/PPE proteins play a critical role in mycobacterial persistence and survival under stress conditions encountered in the host cell, including low pH and higher oxidative stress. The upregulation of PE3 and PE35 has been observed during persistence within the macrophages (6, 9, 10). Recombinant *M. smegmatis* containing PE11 has been reported to enhance survival within the macrophages. Numerous PE/PPE proteins have been shown to modify the cell wall assembly of the *M. tb* (6).

The sophisticated cell wall is a significant source of virulence factors and a principal constituent to survive under extremely harsh conditions encountered in the host cells. The role of these cell wall-modifying proteins in pathogen survival has also been investigated. PE11 has been reported to modify the cell wall’s lipid composition, thereby imparting resistance against various stresses and antibiotics to the recombinant *M. smegmatis* harboring PE11 compared to the control strain (6). PE19 has also been shown to modify the cell wall integrity of *M. tb* and thereby provide resistance to various stress conditions (11). Recently, a novel function of PE/PPE proteins has been deciphered where it has been shown that PE19, together with PPE51, acts as a porin to transport small solutes, i.e., glucose, glycerol, and propionamide. It has also been shown that PE/PPE pair PE20 and PPE31 acts as a transporter to Mg^2+^. Furthermore, the role of PE19–PPE25 and PE32–PPE65 in the transport and acquisition of inorganic phosphate has been reported. Although *M. tb* lacks canonical porins, it is hypothesized that these PE/PPE proteins function analogous to porins to transport small molecules and solutes (12, 13).

PE/PPE proteins are usually surface localized, thus possibly at the interface of host–pathogen interaction. In turn, host cells exploit various innate immune pattern recognition receptors to recognize the pathogen or antigens *via* the pathogen-associated molecular motifs to mount an effective immune response against the invaders (14). In the case of *M. tb*, PE/PPE proteins are known to be recognized by (Toll-Like-Receptors) TLR4, TLR2, TLR2 in combination with TLR1 or TLR6 and modulate downstream signaling cascade to generate and mount an effective immune response (15). Further, there are numerous reports of modulation of cell death pathways. PE25–PPE41 complex and PE_PGRS33 [polymorphic GC-rich-sequence (PGRS)] are known to induce robust necrosis of the infected macrophages, leading to disseminating the disease (16, 17). Intriguingly, PE_PGRS33 is also known to induce enhanced apoptosis *via* TLR2/ASK1/p38 or JNK-MAPK pathways (17). The PE9–PE10 complex is known to interact with TLR4 and induce apoptosis in macrophages (18). A critical host defense mechanism, autophagy, is also known to be exploited by bacteria to dampen innate host defenses (19). The *M. tb* PE_PGRS47 has been shown to inhibit autophagy and Major Histocompatibility Complex II (MHC-II) antigen presentation. It thereby inhibits innate and adaptive immunity to prevent bacteria’s clearance by the host cell (20). The enhanced intracellular survival (Eis) protein of *M. tb* suppresses innate immunity by inhibiting autophagy and producing proinflammatory cytokines TNF-α and IL-6 and caspase-independent cell death through a redox-dependent mechanism. RipA, a secretory endopeptidase, dampens both autophagy and apoptosis in macrophage cells for intracellular survival and virulence (21, 22). *M. tb* is known to inhibit autophagy by suppressing autophagosomes’ fusion to lysosomes. Interestingly, avirulent strains such as H37Ra and BCG lack this feature. This autophagy inhibition by *M. tb* is dependent on ESX-1 type VII secretion and secreted effector ESAT6 protein (23). Moreover, a recent study suggested that two of the anti-*M. tb* drugs isoniazid and pyrazinamide promote autophagy to potentiate anti-TB potential (24). Apart from modulating autophagy, several of these PE/PPE proteins localize to the host organelles. The PE_PGRS protein PE_PGRS5 localizes to endoplasmic reticulum (ER), whereas PPE protein PPE37 is targeted to the nucleus (25, 26). The PE/PPE family proteins are also known to skew the balance between the Th1 and Th2 T-cell immune responses. We have earlier reported that co-operonic PE32–PPE65 hamper the production of Th1 response (27). Biophysical investigations revealed that this co-operonic interaction involves disorder-to-order transition that impacts inflammatory cytokines production (28).

Here we report the role of PE6 in the regulation of proinflammatory cytokine production through the innate immune receptor TLR4. It induces increased production and membrane organization of TLR4, a hallmark of its activation. PE6 also activates the NFĸB signaling cascade downstream to the TLR4 receptor to maintain a sustained inflammatory response. We demonstrate its spatiotemporal localization into nucleus/nucleolus and mitochondria. Using recombinant *M. smegmatis* containing *pe6*, we show that PE6 controls the expression of Nucleolin, indicating possible exploitation of Nucleolin functions to dampen host defenses. We also unravel the opposite role of PE6 in modulating the host cell death pathways, i.e., apoptosis and autophagy. We discerned its role in acquiring iron and enhanced intracellular survival inside macrophages besides possessing DNA binding activity. It appears that PE6 is involved in regulating host–pathogen interaction by regulating multiple host cell functions.

## Materials and Methods

### Generation of Recombinant Constructs

*pe6* gene was cloned into pET-28a and pST-Ki vectors using bacterial artificial chromosome clone (a kind gift from Vinay K. Nandicoorie, NII, New Delhi, India) as a starting material. *E. coli* and *M. smegmatis* strain and PCR primers used in the amplification of *pe6* and PCR conditions are provided in **Tables S1**, **S2**. *pe6* cloned into pET-28a was used to express the recombinant protein in ClearColi^®^ BL21(DE3), an endotoxin-free strain (Lucigen, Middleton, WI, USA). The protein was purified as described earlier with minor modifications (28). Briefly, recombinant PE6 protein was expressed and purified from BL21 DE3 clear *E. coli* cells, induced with 0.5 mM Isopropyl ß-D-1-thiogalactopyranoside at 37°C for 4 h till OD_600_ nm was reached between 0.4 and 0.6. The protein was purified in Tris–NaCl buffer containing 0.3% N-lauryl sarcosine using Ni-NTA (Ni-Nitriloacetic acid) affinity chromatography. The purified protein was dialyzed overnight against the dialysis buffer (10 mM Tris–HCl pH 8.0, 150 mM NaCl, 10% glycerol, 0.001% sarcosyl). The purity of the dialyzed protein was checked using SDS-PAGE and Western blot analysis (**Figures S3A, B**). Endotoxin contamination was removed using Polymyxin B agarose beads, as described earlier. Briefly, an equal amount of protein with polymyxin agarose beads was mixed, left for 2 h for binding on rotating shaker, and centrifuged at 3,000 rpm for 3 min. The supernatant was removed, and protein concentration was estimated. Endotoxin contamination was checked using Limulus amoebocyte lysate, as reported earlier (29) (Thermo Fisher Scientific, USA). *pe6* cloned into the pST-Ki shuttle vector was electroporated into *M. smegmatis*, and expression was confirmed using the anti-Flag antibody (**Figure S3C**) (Sigma, USA). Positive clones were selected, grown, and used for further experiments.

### Cell Culture and Growth Condition

Murine macrophage cell line RAW264.7 and knockout cell lines ΔTLR1, ΔTLR2, ΔTLR4, ΔMyd88, and ΔMyd88/Trif were procured from The Biodefense and Emerging Infections Research (BEI) resources (NIAID, NIH), USA and grown in DMEM media (Gibco, USA) supplemented with 10% Fetal Bovine Serum (Gibco) along with 1% antibiotic-antimycotic solution (Gibco, USA). Cells were grown in standard tissue culture conditions at 37°C in a 5% CO2 incubator.

### Estimation of Cytokines

RAW264.7, ΔTLR1, ΔTLR2, ΔTLR4, ΔMyd88, and ΔMyd88/Trif cells were seeded in a 12-well cell culture plate at a density of 1 million cells/well. Cells were kept at 37°C for 3 h for adherence. After adherence at the bottom of the culture plate, cells were treated with various concentration of the protein, i.e., heat-inactivated protein (HI) (2 µg/ml), 0, 0.5, 1 and 2 µg/ml for 24 h. Proteinase K (Invitrogen, USA) treated followed by the heat-inactivated protein at 2 µg/ml was used as a negative control. Lipopolysaccharide (LPS) (Sigma, USA) at 1 µg/ml was used as a positive control. Culture supernatant was collected and stored at −80°C until further use. Estimation of both proinflammatory (TNF-α, IL-6, and IL-12) and anti-inflammatory cytokines (IL-10) were performed using a mouse ELISA kit provided by BD Biosciences (BD Biosciences, San Jose, USA). The detection limits of ELISA kits used for estimation of TNF-α, IL-6, IL-12, and IL-10 are 15.6, 15.6, 15.6, and 31.3 pg/ml. The protocol provided by the manufacturer was followed. Cytokine levels were determined by measuring absorbance at 450 nm using an ELISA plate reader. Cytokine estimation (TNF-α and IL-6) from recombinant *M. smegmatis* (*pe6* containing *M. smegmatis* and vector alone containing cells) infected macrophage cells were performed from the supernatant collected after 24 and 48 h post-infection. Three million macrophage cells were seeded in 6-well culture plates and left for adherence overnight. Cells were infected with *M. smegmatis* containing *pe6* and vector alone harboring cells for 24 and 48 h at Multiplicity of Infection) MOI 1:10). The supernatant was collected and stored at −80°C until used.

### Cell Death Assay and Surface Expression of TLR4 on Differentiated THP1 Cells

RAW264.7 cells were seeded in 24-well plates at a density of 0.5 million cells/well. After 3 h of adherence, cells were treated with 5, 7.5, and 10 µg/ml of PE6 protein, and incubated for 24 h. Approximately 0.1 µM staurosporine (Sigma, USA) was used as a positive control. Approximately 20 µM pan-caspase inhibitor, ZVAD-FMK (MP Biomedicals, USA), was used as a negative control. Annexin V/PI staining was performed using a BD FACS apoptosis staining kit as per manufacturer protocol. Surface expression of TLR4 on Phorbol 12-myristate 13-acetate (PMA) differentiated (20 ng/ml of PMA for three days) THP1 cells was determined using (0.5 million) cells treated with different concentrations of PE6 (1 and 2 µg/ml). Cells were seeded in a 24-well culture plate. After differentiation, cells were treated with PE6 and harvested after 24 h and incubated with anti-human PE-TLR4 antibody. The samples were processed as per protocol provided by the supplier (BD Biosciences, San Jose CA, USA). LPS (500 ng/ml) treated cells were used as a positive control for TLR4 expression. Fluorescence intensity was measured using the BD FACSVerse cytometer (BD Biosciences). Untreated and heat HI PE6 treated samples were taken as negative controls. The data were analyzed by Flow Jo software (Tree Star Inc. USA).

### Docking of Modeled PE6 to the Ligand-Binding Pocket of TLR4

The modeled PE6 was subjected to protein-protein docking with TLR4, PDB id:3vq2 using the ClusPro docking engine (29, 30). The highest-ranked model based on balanced scores from electrostatic, hydrophobic, and Vander Waals parameters was taken to analyze protein-protein interaction analysis by PDBPisa (31).

### Western Blot Analysis

RAW264.7 cells and ΔTLR4 macrophage cells were seeded at a density of 3 million cells/well in a 6 well culture plate. Cells were treated with 1 and 2 µg/ml concentrations of PE6 protein. HI, protein-treated cells, and untreated cells were used as negative controls. After 24 h of treatment, cell culture media was removed, and cells were washed with 1× cold PBS (Gibco, USA). The cells were harvested in 2× SDS sample loading dye (25% glycerol, 0.125 M Tris–HCl, pH 6.8, 5% SDS, 0.1% bromophenol blue, with 100 mM dithiothreitol added fresh each time). Samples were heated at 95°C for 10 min, cooled, and pulsed centrifuged to remove cell debris. An equal amount of protein was loaded in SDS-PAGE gel and transferred onto the PVDF membrane. The transferred blot was blocked using BSA (5%) or skimmed milk (5%) in TBST and probed against NFκB1 (Cloud-Clone Corp), pP-65, (Thermo Fisher Scientific, USA), Caspase 3, Caspase 9, Bax, Cytochrome C, pcMyc, LC3BII, p62/SQSTM1, Beclin1, pULK1, MtorC1, Atf6, Chop, Calnexin, BIP, Eif2α, Ire1α, Nucleolin (Cell Signaling), GroEL2, Mpt32 (BEI Resources, USA), GAPDH and β-actin antibodies (Santa Cruz Biotechnology) as per manufacturer’s protocol. The anti-PE6 antibody was used to study the localization of PE6 in various *M. tb* fractions. anti-Flag (Sigma, USA) and anti-His (Sigma, USA) antibodies were used to detect recombinant PE6.

Horse Radish peroxidase-conjugated secondary antibodies (anti-mouse or anti-rabbit) were used for signal generation (Sigma, USA). In order to study the autophagy pathway, cells were treated with rapamycin (200 nM) and bafilomycin A1 (50 nM) (Sigma, USA) for 6 h along with protein treatment (24 h for Western blot analysis, 24 and 48 h for immunofluorescence analysis of LC3BII punctate foci formation). Cells were harvested and processed, as described earlier, followed by Western blot analysis. BSA (5%) was used for blocking in the Western blot of phosphoproteins. Images were captured using the Bio-Rad-Chemi-Doc MP imaging system (Bio-Rad Laboratories India Pvt. Ltd.). The band intensity was quantified using ImageJ software and normalized to GAPDH or β-actin.

### Generation of Anti-PE6 Antibody in Rabbit

Purified PE6 protein was used as an antigen to generate an anti-PE6 antibody. Approximately 500 µg of purified protein was mixed with an equal volume of Freund’s incomplete adjuvant (500 µl each) and emulsified. Produced protein adjuvant emulsion was used for injection into 90–120 days old New Zealand white rabbit. Three booster doses were given at an interval of 21 days. After the final booster dose rabbit was bled, and serum was collected and stored at −80°C until used. The specificity of the anti-PE6 antibody was validated using Western blot analysis (**Figure S3D**).

### Infection of Recombinant *M. smegmatis* Containing *pe6* Into RAW264.7 Cells

RAW264.7 cells were seeded at a density of 2 million cells/well in a 6-well culture plate and incubated overnight for adhesion. Log phase growing *pe6* and vector alone transformed *M. smegmatis* were collected and washed with 1× PBS. A single-cell suspension was prepared by passing the bacteria through a 24-gauge syringe needle. The prepared single-cell suspension of recombinant *M. smegmatis* was used for infection at an MOI of 1:10 for 24 to 48 h. To determine colony-forming units (CFU) and intracellular survival, RAW264.7 cells were lysed in sterile 0.01% SDS prepared in 1× PBS and plated on 7H11 agar plates (BD, Difco, USA). Colonies were counted after four days of incubation at 37°C. For Western blot analysis, cells were washed with 1× cold PBS and harvested in 2× SDS sample loading dye. An equal amount of protein was loaded in SDS-PAGE, and protein was transferred onto the PVDF membrane, blocked, and probed with desired antibodies.

### Immunofluorescence and Confocal Laser Microscopic Analysis

HEK293T and macrophage cells were grown on coverslips in a 24-well plate for microscopic analysis. HEK293T cells were transiently transfected with pEGFPN1-PE6 (GFP-PE6) and pEGFPN1 (GFP), using lipofectamine 3000 (Thermo Fisher Scientific, USA). After 24, 48, and 72 h post-transfection, cells were either stained with Mitotracker Deep Red FM (Thermo Fisher Scientific, USA) as per manufacturer’s protocol or with an anti-Nucleolin antibody (Cell Signaling, USA). Cells were fixed with 4% formaldehyde in 1× PBS, followed by permeabilization with 0.2% Triton-X100 in PBS. For nucleolar localization of PE6, manufacturers protocol was followed for staining the cells with the anti-Nucleolin antibody. RAW264.7 cells were treated with PE6 protein at a concentration of 1 and 2 µg/ml. After 24 h of treatment, cells were fixed and permeabilized with chilled methanol for 5 min, followed by washing with 1× PBS. Coverslips were incubated with primary antibodies (NFκB1, pP65, and TLR4) as per the manufacturer’s protocol. For studying the surface localization of PE6 in RAW264.7 cells, PE6 protein was treated at 2 µg/ml, after 2 h of treatment, cells were fixed using 4% formaldehyde, washed, and treated with anti-PE6 antibody (1:500) for overnight at 4°C. For studying the effect of PE6 on autophagy using RAW264.7 cells, a similar immunofluorescence protocol was followed as described. Anti-rabbit LC3 antibody (Cell signaling, USA) was used to detect membranous punctate foci. DAPI (Sigma, USA) was used to stain the nucleus. Cells were washed and incubated with Alexa fluor 488 (A488) or A547 conjugated secondary antibodies for 2 h. The coverslips were mounted using Prolong anti-fade mounting agent (Thermo Fisher Scientific, USA), viewed at 63× using a Carl Zeiss fluorescence microscope and a Olympus FLUOVIEW FV1000 confocal laser scanning microscope. Images were analyzed using Axio-vision and FLUOVIEW FV1000 software. Spatiotemporal localization of PE6 at 24, 48, and 72 h were presented from 100 positively transfected cells from three independent experiments. Punctate foci representing LC3BII were analyzed using Axio-vision software (Carl Zeiss, Germany) from 10 different fields and the data presented are representative count from 50 cells.

### Co-Immunoprecipitation (Co-IP)

HEK293T (2 million) cells were cultured in 6-well tissue culture plate transfected with pEGFP-N1 and pEGFP-N1-PE6 for 24 h, re-suspended in 750 µl of lysis buffer (1× PBS pH 7.4, 2 mM EDTA, 2 mM DTT, 1% TritonX-100, 1mM PMSF, and protease inhibitor cocktail, Sigma-Aldrich, USA), lysed by sonication [three cycles (15 s each at 2 min cooling interval) at an amplitude of 20%], centrifuged at 13,000 rpm for 30 min. The supernatant was pre-cleared by adding pre-immune rabbit serum, along with protein-A agarose beads. Approximately 750 μl of pre-cleared samples (750 µg/ml total protein) was used in Co-IP. To each pre-cleared sample, 3 μl of anti-PE6 and 1:50 dilution of anti-Nucleolin antibodies were added, incubated on a rotating rocker overnight at 4°C. Next, 25 μl of the packed volume of protein-A agarose beads were added to each sample, incubated for an additional 2 h, centrifuged at 3,000 rpm for 1 min, the supernatant was discarded, and the beads were washed with lysis buffer (5 min each washing on rotating shaker at 4°C). Bound protein complexes were released by boiling the beads in 2× SDS sample loading buffer and then subjected to SDS-PAGE followed by Western blot analysis using the desired antibodies.

### Survival Assay Under the Low Iron Condition

Recombinant *M. smegmatis* cells harboring *pe6* and vector alone were grown in 7H9 media (BD Difco, USA) supplemented with 10% OADC (Himedia, India) till the O.D reached 0.6. The culture was then diluted to an O.D to 0.1. The media was supplemented with 150 µM 2,2’- Bipyridyl (Sigma, USA), used as an iron-chelating agent to deplete the iron. To supplement the iron 150 µM Fecl3 was added in the presence of BPS. The culture O.D was taken every 3 h till O.D reaches 1. Wild-type, *pe6*, and vector alone containing *M. smegmatis* cells were diluted to an O.D of 0.1. A 10-fold serial dilution was made and spotted onto a 7H11 agar plate containing no BPS, 150 µM BPS, and 150 µM BPS, or 150 µM BPS + 150 µM Fecl3. The growth pattern was observed after four days of incubation at 37°C.

### Computational Analysis of PE6 Sequence Information

Mitochondrial localization signal sequence in PE6 was predicted using MITOPROT (https://ihg.gsf.de/ihg/mitoprot.html), TargetP 1.1 (http://www.cbs.dtu.dk/services/TargetP/) and PSORT II (https://psort.hgc.jp/form2.html). The presence of a nucleus targeting sequence in PE6 was analyzed using LocSigDB.

### DNA and Iron-Binding Activity Analysis of PE6

Full-length PE6 was modeled using i-Tasser (32). The resultant model was checked for quality using ProSA and Procheck analysis (33, 34). DNA binding capability of PE6 was determined using the DNABind server. DNA-protein modeling was performed using the NP-Dock program (35). *In vitro* DNA binding assays were carried out using steady-state fluorescence titrations. Briefly, 2 µM of PE6 protein was titrated against increasing concentrations of dsDNA (up to 10 µM). All experiments were performed in 10 mM Tris–HCl pH 7.4, 100 mM NaCl at 298 K in a Perkin Elmer LS55 spectrofluorometer. The excitation wavelength was set at 290 nm, while the emission spectrum was recorded between 300 and 450 nm. Buffer controls were recorded by titration of protein with buffer. The single site-specific binding module of GraphPad (v5.0) was used to determine Kd from fluorometric titrations data using the following equation:

Y=Bmax∗X/(Kd+X)

where Bmax is maximum binding upon addition of ligand X (dsDNA).

To evaluate the thermal stability of PE6 in the presence of dsDNA, an intrinsic fluorescence-based thermal shift (FTS) assay was performed by monitoring change in fluorescence emission at 330 nm with gradual temperature ramping from 25 to 90°C (2°C step size). Excitation and emission slits were kept at 5/10. Before each reading, an equilibration time of 1 min was kept for both PE6 (4 μM) alone and 10 μM dsDNA. PE6 was pre-incubated with 10 μM of DNA for 30 min at 298K before experiments.

### Statistical Analysis

Data were analyzed using one-way ANOVA with Tukey’s multiple comparisons, two-way ANOVA with Sidak’s multiple comparisons, paired T-test in combination with Kruskal–Wallis post-test. The data were represented as the mean of replicates (mean from three independent or two independent experiments ± SD. P values <0.05 were considered as significant. Data analysis was carried out using GraphPad Prism 7.

## Results

### PE6 Is a Secreted and Surface Localized Protein of *M. tb* That Induces Robust Production of Proinflammatory Cytokines in Macrophages

**Figure 1:**
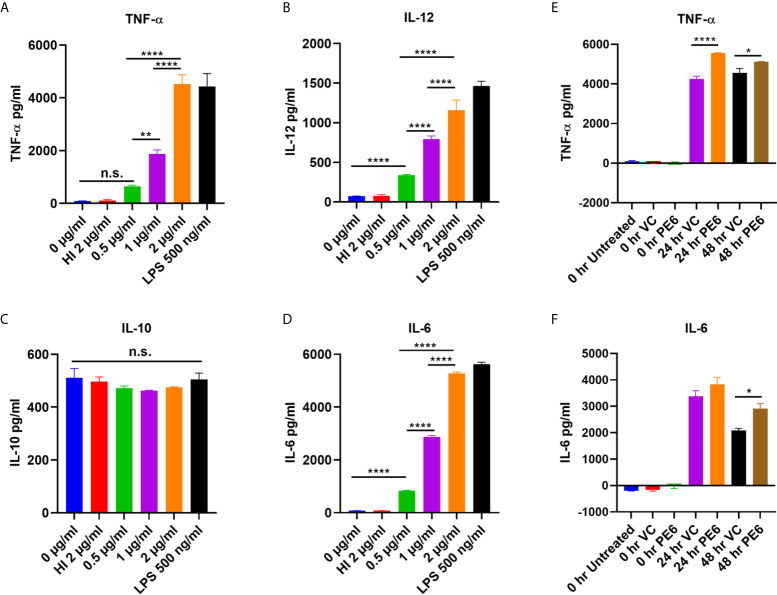
PE6 is a proinflammatory molecule of *M. tb*, which induces robust secretion of proinflammatory cytokines. **(A–D)** RAW264.7 macrophage cells were treated with various concentration of PE6 (0.5, 1 and 2 μg/ml), LPS 500 ng/ml and HI PE6 (2 μg/ml). Sandwich ELISA was used to quantify cytokine TNF-α, IL-6, IL-12, and IL-10 levels using the cell culture supernatants. LPS treatment was used as a positive control for the induction of proinflammatory cytokines. Untreated and HI PE6 treated cells were used as negative controls. Purified PE6 was treated with polymyxin B agarose beads for the removal of endotoxin, and one aliquot was digested with proteinase K, followed by heat inactivation at 100 °C for 4 h. **(E, F)** Three million RAW264.7 cells were seeded in a six-well tissue culture plate and left overnight for adherence. Cells were infected with recombinant *M. smegmatis* containing *pe6* and vector alone cells at (MOI 1:10) for 24 and 48 h. TNF-α and IL-6 levels were measured using sandwich ELISA. Untreated and 0 h treated cells were used as negative controls. Data were analyzed by one-way ANOVA with Tukey’s multiple comparison post-test. Data are representative of three independent experiments. P values <0.05 were considered as significant. *P < 0.05, **P < 0.01 and, ****P < 0.0001 *vs*. controls. n.s., not significant.

PE6 of *M. tb* is a cell surface-associated protein found in the cell wall (36). Proteins present in the cell envelop interact with the host to modulate immune responses. PE6 is localized in multiple locations in *M. tb*, as evidenced by analysis of various cell fractions using Western blot analysis. Various *M. tb* cell fractions were separated by SDS-PAGE, transferred onto the PVDF membrane, and probed with an anti-PE6 antibody. PE6 was detected in various fractions, including cell-wall, total membrane, and culture filtrate (**Figure S1**). Localization of GroEL2 and Mpt32 was used as controls for cell wall, cytosol, and secretory fractions. Therefore, we investigated the production of proinflammatory and anti-inflammatory cytokines after PE6 treatment to macrophages. RAW264.7 cells were treated with a different non-toxic concentration of PE6 protein (0.5 to 2 µg/ml). RAW264.7 cells upon treatment with PE6 induced increased production of proinflammatory cytokines TNF-α, IL-6, and IL-12 in a dose-dependent manner, whereas no significant difference was observed in the production of anti-inflammatory cytokine (IL-10 secretion) (**Figures 1A–D**). A batch of protein was proteinase K digested and heat-inactivated every time and added to macrophages to rule out non-specific or LPS mediated activation of cytokines, even though every batch of protein was passed through polymyxin agarose for removal of endotoxin. To confirm our initial observations, we generated a knock-in strain of recombinant *M. smegmatis* containing *pe6* and used vector-alone transformed cells as control. RAW264.7 cells were infected with similar colony-forming units (MOI 1:10) for 24 h, and 48 h with recombinant *M. smegmatis* containing *pe6* and vector alone transformed cells proinflammatory cytokines levels (TNF-α, and IL-6) were measured using ELISA. We observed that *pe6* containing recombinant *M. smegmatis* induced enhanced secretion of TNF-α and IL-6 (**Figures 1E, F**). These observations suggest that PE6 is a cell surface localized and secreted protein of *M. tb* involved in the robust production of proinflammatory cytokines.

### PE6 Interacts With Innate Immune Receptor TLR4 and Induces Its Enhanced Production on Macrophage Cells

**Figure 2:**
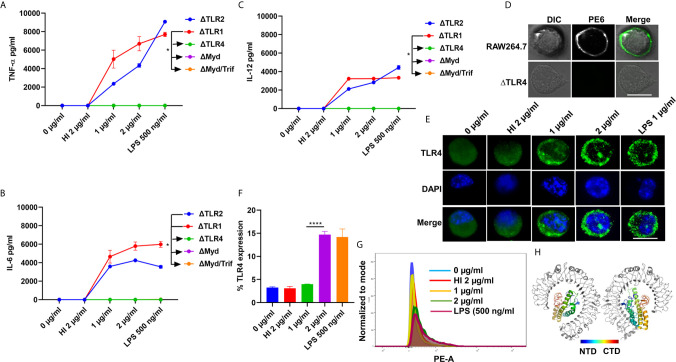
PE6 interacts with surface immune receptor TLR4 and induces its increased expression and secretion of proinflammatory cytokines. **(A–C)** Mouse macrophage knockout cells ΔTLR1, ΔTLR2, ΔTLR4, ΔMyd88, and ΔMyd88/Trif, were stimulated with PE6 (1 and 2 μg/ml), LPS 500 ng/ml and HI PE6 (2 μg/ml) treated with proteinase K (PK; 50 mg/ml), followed by heat inactivation at 100°C for 4 h. Approximately 24 h of post-treatment, cell culture supernatants were collected, and sandwich ELISA quantified TNF-α, IL-6, and IL-12 levels. **(D)** RAW264.7 and ΔTLR4 macrophage cells were treated with purified recombinant PE6 (2 µg/ml). Approximately 2 h of post-treatment, cells were fixed using 4% formaldehyde to stabilize the interaction of PE6 to the cell surface localized receptor. Fixed cells were washed and probed with polyclonal anti-PE6 antibody (1:500) dilution for 2 h. Post-primary antibody treatment, cells were washed three times with cold PBS and probed with anti-rabbit A488 conjugated secondary antibody. After three washing steps with cold PBS, cells were mounted on ProLong Anti Fade Glass mount and visualized using Carl Zeiss fluorescence microscope. Scale bar indicates 10 µm. **(E)** Immunofluorescence microscopic images showing membrane organization and expression pattern of TLR4 in PE6 treated macrophage cells. Concentrations of PE6 used are marked in the figure. The anti-rabbit TLR4 monoclonal antibody was used to probe the localization of TLR4. A488 conjugated secondary antibody was used for signal detection. DAPI was used to stain the nucleus. Untreated and HI PE6 treated cells were used as negative controls. LPS treated cells were used as a positive control. Scale bars indicate 10 µm. **(F)** The quantification of TLR4 expression is shown as a bar graph. **(G)** FACS was used to study the expression of TLR4 on PMA differentiated and PE6 treated THP1 cells. Monoclonal anti-TLR4 antibody conjugated to PE fluorophore was used in the FACS experiment. Data were analyzed by one-way ANOVA with Tukey’s multiple comparison post-test. Data are representative of three independent experiments. *P < 0.05 and ****P < 0.0001 *vs*. controls. **(H)** TLR4-PE6 model of the top-scoring structure obtained through ClusPro protein-protein docking. PE6 (Colored) protein fits into TLR4 (Gray) ligand-binding pocket. The colored bar indicates coloring from N-terminal to the C-terminal domain of PE6.

Pathogen-associated molecular patterns (PAMPs) are recognized by various TLRs present on the host cell surface. *M. tb* and its associated antigens are recognized by surface innate immune receptors TLR2 and TLR4. We confirmed the role of TLR4 in the production of proinflammatory cytokines in response to PE6. We treated various concentrations of PE6 (1 µg and 2 µg/ml) to the ΔTLR1, ΔTLR2, ΔTLR4, ΔMyd88, and ΔMyd88/Trif knockout cells and measured proinflammatory cytokines (TNF-α, IL-6, and IL-12) levels using sandwich ELISA. We found that PE6 robustly induces the production of proinflammatory cytokines by ΔTLR2 and ΔTLR1 cells. In contrast, ΔTLR4, ΔMyd88, and ΔMyd88/Trif cells produced negligible amounts of these cytokines, indicating that PE6 interacts and activates TLR4 and downstream adaptor protein Myd88 for the production of proinflammatory cytokines (**Figures 2A–C**). These observations suggest that the absence of TLR4 on the macrophages’ surface impaired their ability to recognize PE6 and was unable to induce proinflammatory cytokines. These results indicate that TLR4 is the innate immune receptor involved in recognizing this mycobacterial protein and may affect the downstream signaling cascade emanating from TLR4 (**Figures 2A–C**).

To further validate the innate host receptor involved in recognizing PE6 and the downstream signaling, we treated wild-type RAW264.7 cells and ΔTLR4 knockout macrophage cells with recombinant purified PE6 protein (2 µg/ml). After treatment, recombinant PE6 protein was fixed on the cell surface together with its interacting receptor. Its localization was observed using fluorescence microscopic analysis. We found that PE6 is localized on the cell surface in RAW264.7 macrophage cells, whereas no signal was observed in ΔTLR4 knockout cells indicating that PE6 interacts with TLR4 on the macrophage cell surface (**Figure 2D**). Consistent with this finding, treatment of PE6 to RAW264.7 and PMA differentiated human-derived THP1 cells induced increased production and organization of TLR4 on the cell membrane, which is a hallmark of its activation (**Figures 2E–G**). Additionally, to confirm the interaction of PE6 to TLR4, we used modeled PE6 for protein docking. The PDBPisa analysis of the protein–protein interaction interface revealed that N-terminal (a.a between 9 and 67) and C-terminal domains (a.a between 146 and 161) of PE6 interact with the ligand-binding pocket of TLR4 (**Figure 2H** and **Table S3**). The interactions were majorly mediated through Hydrogen-bond interactions followed by salt bridges (**Table S3**). These findings suggest that PE6 is a specific TLR4 agonist that induces robust secretion of proinflammatory cytokines.

### PE6 Activates the Canonical NFĸB Pathway

**Figure 3:**
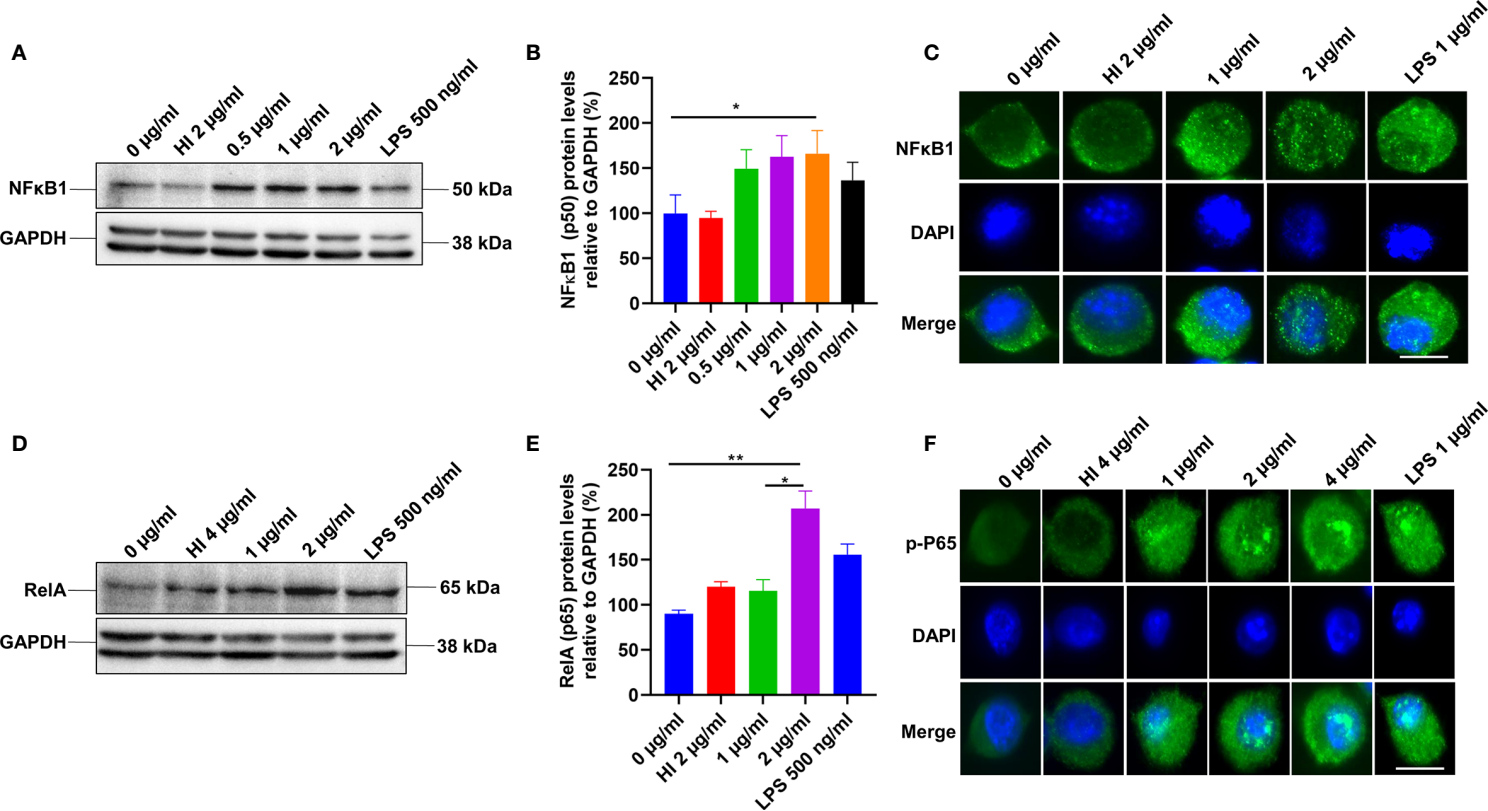
PE6 induces the activation of the canonical NFκB signaling pathway. **(A)** Western blots showing the expression level of NFκB1 (P50) subunit of NFκB in PE6 treated RAW264.7 macrophage cells. RAW264.7 cells were treated with PE6 (1 and 2 µg/ml) for 24 h. **(B)** Densitometric quantitation of the P50 subunit of canonical NFκB. P50 protein levels were expressed relative to GAPDH [%]. **(C)** Immunofluorescence microscopic pictures showing nuclear translocation of P50 subunit. **(D)** Western blots showing the protein levels of the phosphorylated pP65 (RelA) subunit of NFκB in PE6 treated RAW264.7 macrophage cells. **(E)** Densitometric quantitation of pP65 subunit. The pP65 protein levels were expressed relative to GAPDH [%]. **(F)** Immunofluorescence microscopic pictures showing nuclear localization of phosphorylated P65. DAPI was used to mark the nucleus. Untreated and HI PE6 treated cells were used as negative controls. LPS (500 ng and 1 µg/ml) treatment was used as a positive control. GAPDH was used as a loading control. A488 linked secondary antibody was used for signal detection. Scale bars indicate 10 µm. Data were analyzed by one-way ANOVA with Tukey’s multiple comparison post-test. Data are representative of three independent experiments. *P < 0.05 and **P < 0.01 *vs*. controls.

TLR4 receptor activation on the macrophages’ surface leads to the recruitment of Myd88 and activation of the NFκB pathway (37). NFκB is a vital transcription factor whose activation and nuclear translocation are essential for producing proinflammatory cytokines by host macrophages. Therefore, we analyzed the expression and activation of canonical NFκB sub-units P50 and pP65 in the presence of PE6 protein using Western blot and immunofluorescence microscopic analysis. RAW264.7 cells were treated with purified recombinant PE6 protein (0 µg/ml, 0.5 µg to 2 µg/ml), and expression and nuclear localization of the P50 subunit were analyzed. PE6 induced increased expression of the P50 subunit, as evident from Western blot analysis (**Figures 3A, B**). Immunofluorescence microscopic analysis showed that PE6 activated its nuclear translocation in a dose-dependent manner (**Figure 3C**). To confirm these findings for another subunit, P65, we employed Western blot and immunofluorescence microscopic analysis. PE6 treatment-induced activator phosphorylation (Ser536) and nuclear translocation of pP65 in a dose-dependent manner, indicating its activation status (**Figures 3D–F**). P50 and P65 subunits form the most common heterodimeric complexes during activation of the canonical NFĸB pathway. P65 subunit is also activated by phosphorylation, which is mediated by kinase inhibitor of nuclear factor kappa-B kinase subunit beta (38). Phosphorylation plays a critical role in the activation of the P65 subunit and NFĸB. Together, our findings suggest that PE6 activates the canonical NFĸB signaling pathway that mediates proinflammatory cytokines’ sustained production.

### PE6 Displays Time-Dependent Differential Localization Pattern in Transiently Transfected HEK293T Cells

**Figure 4:**
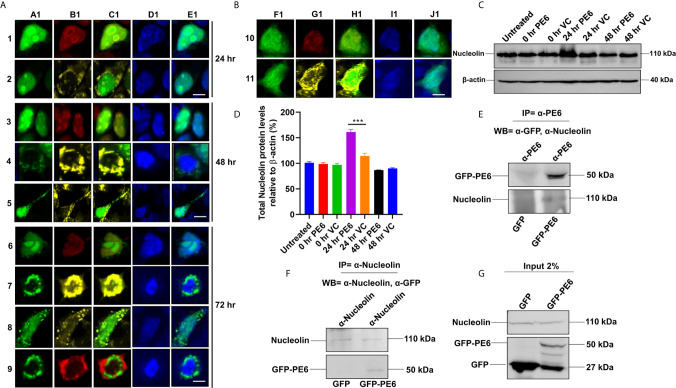
*M. tb* PE6 targeted to nucleus/nucleolus and mitochondria in transiently transfected HEK293T cells interacts with Nucleolin. **(A, B)** Confocal laser scanning microscopic analysis of GFP and GFP-PE6 expressing HEK293T cells. Transiently transfected cells were fixed using 4% formaldehyde and washed with cold PBS. GFP-PE6 (green) and GFP (green) localization was analyzed at 24, 48, and 72 h post-transfection. Nucleolus was stained using an anti-Nucleolin antibody (pseudo red). Mitotracker deep red FM was used to study the mitochondrial localization (pseudo yellow). DAPI was used to stain the nucleus (blue). A547 conjugated secondary antibody was used for signal generation. Scale bars indicate 10 µm. **(C)** Western blots showing the Nucleolin level in macrophage cells infected with recombinant *M. smegmatis* containing *pe6* and vector. After 24 h of infection, cell lysate was prepared and fractionated on SDS-PAGE, and proteins were transferred onto the PVDF membrane. Nucleolin levels were analyzed using the anti-Nucleolin antibody. Untreated and vector alone transformed cells were used as controls. β-actin was used as a loading control. **(D)** Densitometric quantitation of Nucleolin bands was represented as a bar graph. Nucleolin level was normalized to respective β-actin bands and represented as [%] protein levels to β-actin. Data were analyzed by one-way ANOVA with Tukey’s multiple comparison post-test. Data are representative of three independent experiments. ***P < 0.001 *vs*. control. **(E, F)** Western blots showing Co-IP of PE6 and Nucleolin from cell extracts prepared from HEK293T cells expressing GFP and GFP-PE6 using anti-PE6 and anti-Nucleolin antibodies, respectively. Lysates prepared from HEK293T cells expressing GFP were used as a negative control. Antibodies used in Co-IP and the Western blots are marked in the figure. **(G)** Protein levels of Nucleolin, GFP-PE6, and GFP in 2% input used in Co-IP experiments.

It was earlier reported that PE6 contains a mitochondria localization signal. Our *in-silico* analysis revealed that PE6 harbors an N-terminal signal peptide (1–24 aa), a mitochondrial targeting sequence, and a nuclear localization signal (LocSig Database). PE6 also harbors the Spt5 C-terminal nonapeptide repeat binding Spt4 domain. Spt4 and Spt5 together control transcription elongation by RNA polymerase II (**Figure S2**) (39). The presence of signal peptide, together with its cell-wall or surface association and secretory nature, reinforces that PE6 may be utilized by *M. tb* to modulate host–pathogen interaction. It has been earlier reported that various PE/PPE proteins are targeted to specialized host organelles, including mitochondria, nucleus, and endoplasmic reticulum (ER) to hijack or subdue innate host defenses (25, 26, 40, 41). These observations prompted us to study the organellar localization of PE6 as well as its associated functions. We used a transient transfection experiment to determine its organellar localization. Upon expression of GFP-PE6 and GFP into HEK293T cells, its localization was visualized up to 72 h. We observed that PE6 exclusively localizes within the nucleus and nucleolus 24 h post-transfection (100% co-localization) (**Figures 4A**, **1A1–1E1**, **2A1–2E1**). However, as time progressed (48 h), PE6 localization was observed in nucleus/nucleolus (70%), only mitochondria (20%), and nucleus/nucleolus together with mitochondria (10%) (**Figures 4A**, **3A1~3E1**, **4A1–4E1**, **5A1–5E1**). At 72 h post-transfection PE6 localized to nucleus/nucleolus (50%), exclusively to mitochondria (30%), and nucleus/nucleolus together with mitochondria (15%). Interestingly, at 72 h post-transfection, we found PE6 co-localization with Nucleolin in the cytoplasm at the periphery of the cells (5%) (**Figures 4A**, **6A1–6E1**, **7A1–7E1**, **8A1–8E1**, **9A1–9E1**). We used GFP localization as control which was similar in all-time points studied. GFP was diffusely localized throughout the cell, including the nucleus and cytoplasm (**Figures 4B**, **10F1–10J1**, **11F1–11J1**). These results indicate that PE6 follows a spatiotemporal localization in transfected HEK293T cells, which suggested a possible role in regulating multiple organellar functions. PE6 likely modulates the functioning of two vital host cellular organelle to enable the survival of *M. tb* within the host. Next, we investigated the functional relevance of PE6 localization into the nucleus/nucleolus. RAW264.7 cells were infected with recombinant *M. smegmatis* containing *pe6* and vector alone, and the Nucleolin level was analyzed using Western blot analysis. Infection with *pe6* containing *M. smegmatis* to RAW264 cells leads to the increased production of Nucleolin at 24 h post-infection compared to vector alone transformed cells, indicating a time-dependent role played by PE6 in the functional regulation of Nucleolin function (**Figures 4C, D**). These findings prompted us to explore the interaction between PE6 and Nucleolin. To study the interaction between PE6 and Nucleolin, Co-IP was performed on TritonX-100 solubilized HEK293T cells expressing GFP and GFP-PE6 cell extracts using anti-PE6 and anti-Nucleolin antibodies. Co-immunoprecipitated samples were separated in SDS-PAGE, and Western blotting was performed using anti-GFP and anti-Nucleolin antibodies. Co-IP results demonstrate that anti-PE6 and anti-Nucleolin antibodies efficiently precipitated GFP- PE6 and endogenous Nucleolin (**Figures 4E–G**). However, no PE6 protein signals were observed in cell extracts prepared from GFP expressing HEK293T cells, using anti-PE6 and anti-Nucleolin antibodies. Taken together, these results suggest the interactions of PE6 with Nucleolin under physiological conditions.

**Figure 5:**
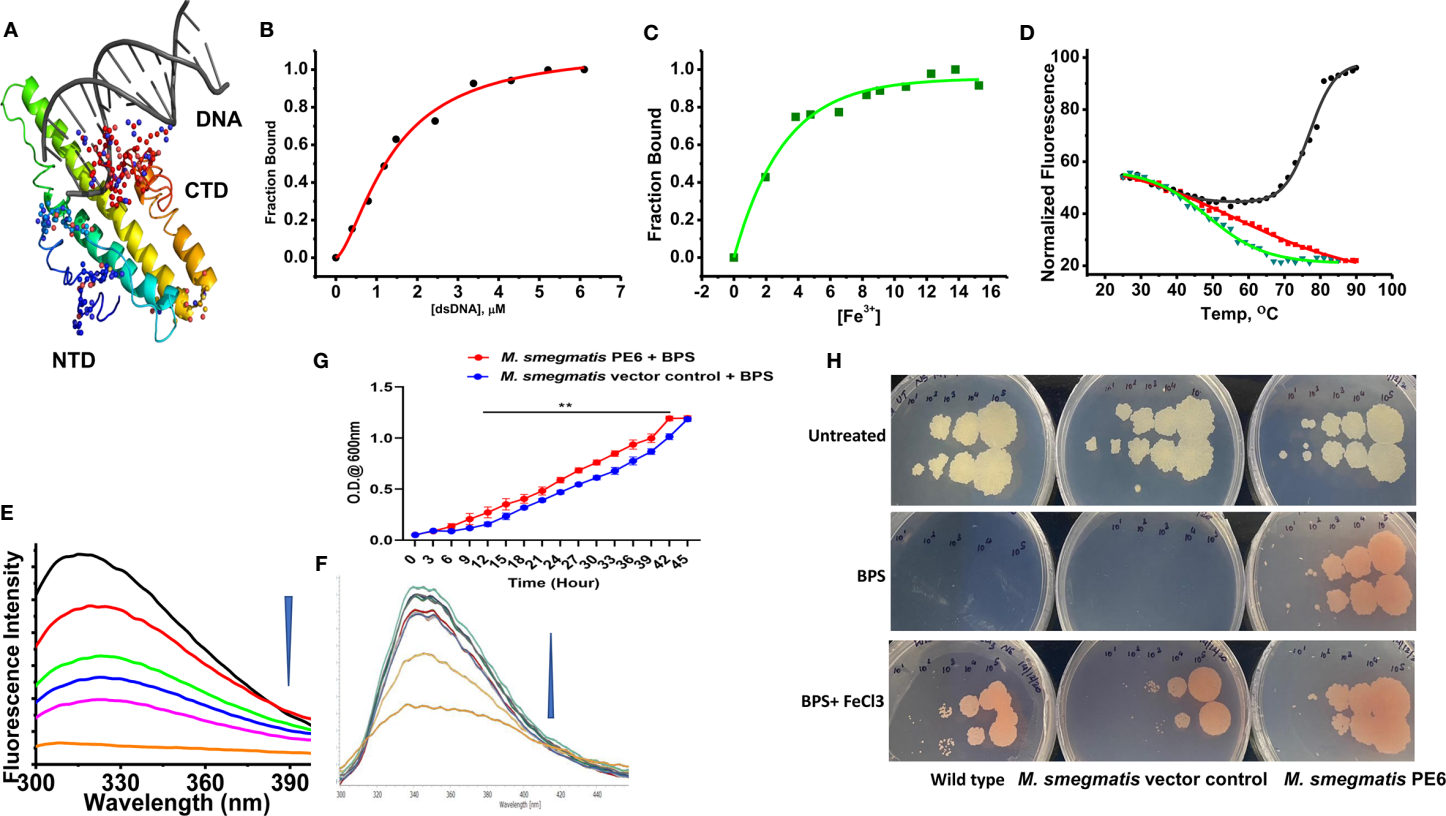
PE6 binds to DNA and iron and *M*. *smegmatis* harboring *pe6* grown in iron-depleted liquid and solid media. **(A)** PE6-DNA modeled structure. Putative DNA binding regions are shown as spheres along with N-terminal and C-terminal domains. **(B–D)** Nonlinear curve fitting analysis to determine the apparent dissociation constant from titrations of PE6 with **(A)** dsDNA, **(B)** Fe. **(C)** FTS assay to determine the melting temperature (Tm) of PE6 alone (Black) and in the presence of dsDNA (Red) and Fe (Green). **(E, F)** Changes in fluorescence emission spectra (300–400 nm) upon the titration of **(E)** dsDNA and **(F)** Fe against PE6. **(G)** Growth kinetics of recombinant *M. smegmatis* harboring *pe6* and vector alone transformed cells in liquid broth containing iron chelator. O.D600 nm was taken at different time intervals, as marked in the graph. *pe6* containing *M. smegmatis* grows significantly faster than vector alone transformed cells. **(H)** The growth pattern of recombinant *M. smegmatis* containing *pe6* and vector alone transformed cells in solid media with and without iron chelator and supplementation with Fecl3. The upper panel shows the growth pattern of untreated wild-type, vector alone, and *pe6* transformed cells, and the middle and the lower panel shows the growth pattern of wild-type *M. smegmatis*, recombinant *M. smegmatis* containing *pe6*, and vector alone transformed cells in the presence of BPS and BPS + Fecl3. Some 10-fold serial dilutions were plated, and growth was observed on the fourth day after plating. Data were analyzed by two-way ANOVA with Sidak’s multiple comparison post-test. Data are representative of three independent experiments. **P < 0.01 *vs*. controls.

**Figure 6:**
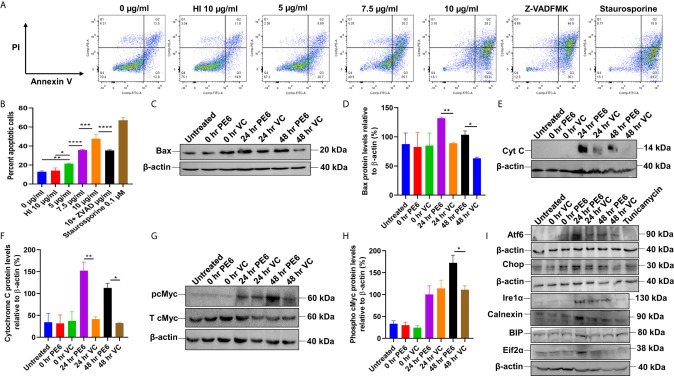
PE6 induced apoptosis activation by inducing pro-apoptotic factors and efficiently activating the ER stress-mediated UPR pathway. **(A)** Flow cytometric analysis of early apoptotic cells of PE6 treated macrophage cells. Untreated and HI PE6 treated cells were used as negative controls. In contrast, staurosporine and ZVAD-FMK treated cells served as positive and negative controls for caspase-dependent induction and apoptosis repression. Briefly, RAW264.7 cells were seeded in a 6-well tissue culture plate, after 2 h of adherence at 37°C, the cells were treated with PE6 (5, 7.5, and 10 µ/ml), HI PE6 (10 µg/ml), 0.1 µM staurosporine, and 20 µM pan-caspase inhibitor Z-VAD-FMK. After completing the treatment, cells were harvested and processed as instructed by the manufacturer (BD Biosciences, San Jose, USA). Sample’s reading was captured using the BD FACSVerse machine. Images were analyzed using FlowJo software. **(B)** Graphical representation of apoptosis induction by treatment of various concentrations of PE6. **(C)** Western blot analysis of recombinant *M. smegmatis* infected macrophage cells after 24 and 48 h of infection. Western blot was performed using an anti-Bax antibody. **(D)** Densitometric analysis of Bax protein levels normalized to β-actin. **(E)** Western blot showing the levels of Cytochrome C protein in macrophage cells infected with recombinant *M. smegmatis*. **(F)** Densitometric quantitation of Cytochrome C protein levels relative to β-actin represented as a bar graph. **(G)** Western blot analysis of pcMYC levels in recombinant *M. smegmatis* infected macrophage cells. **(H)** Densitometric quantitation of pcMYC levels relative to β-actin and represented as a bar graph. Untreated and vector alone transformed cells were used as negative controls. Protein levels were represented as [%] to β-actin and total cMYC. Data were analyzed by one-way ANOVA with Tukey’s multiple comparison post-test. Data are representative of three independent experiments. *P < 0.05, **P < 0.01, ***P < 0.001, ****P < 0.0001 *vs*. controls. **(I)** Western blot analysis showing the levels of UPR markers in recombinant *M. smegmatis* infected macrophage cells. RAW264.7 macrophage cells were infected with recombinant *M. smegmatis* at MOI of 1:10 for 24 and 48 h. The cell lysate was prepared, run in SDS-PAGE, transferred onto the PVDF membrane, and Western blotted using anti-Atf6, anti-Chop, Ire1α, Calnexin, BIP, and Eif2α antibodies. Untreated and vector alone transformed cells were used as controls. β-actin was used as a loading control.

**Figure 7:**
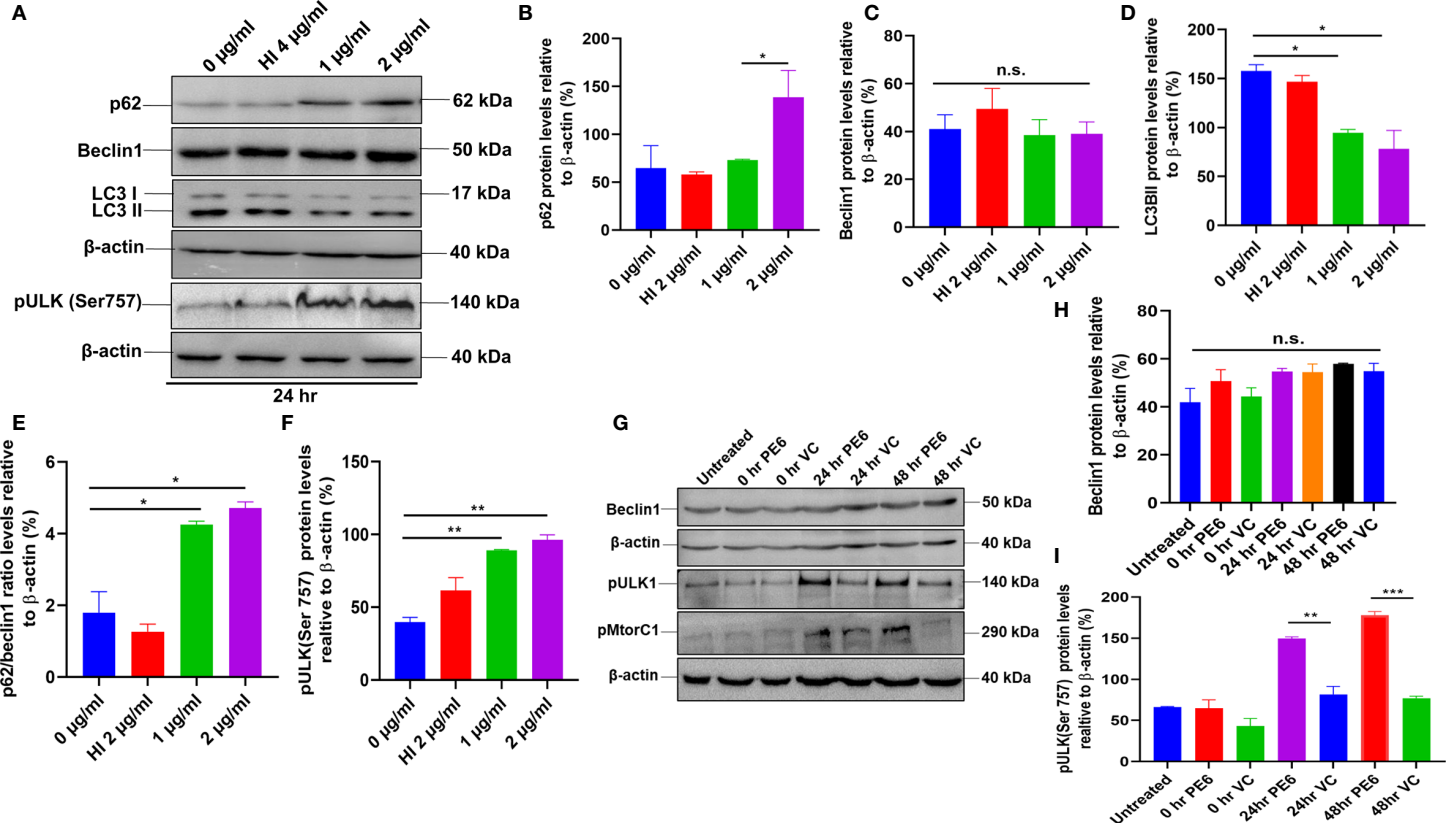
PE6 inhibits autophagy in treated RAW264.7 cells. **(A)** Western blots demonstrating the inhibitory effect of PE6 on cellular autophagy of RAW264.7 macrophage cells utilizing autophagy markers p62, Beclin1, LC3BII, and pULK1. RAW264.7 macrophage cells were treated with different concentrations of PE6 (1 and 2 µg/ml) for 24 h. The cell lysate was prepared, run in SDS-PAGE, proteins were transferred onto the PVDF membrane and probed with indicated antibodies. Untreated and HI-treated cells were used as negative controls. **(B–F)** Densitometric quantification of the protein bands (p62, Beclin1, LC3BII, and pULK1, and the ratio of p62/Beclin1) are shown. Protein levels were represented as [%] to β-actin. **(G)** Western blot demonstrating the levels of autophagy markers Beclin1, pULK1, and pMtorC1 after infection with recombinant *M. smegmatis* to RAW264.7 macrophage cells at MOI of 1:10 for 24 and 48 h. β-actin was used as a loading control. Uninfected and vector-alone transformed cells were used as negative controls. **(H, I)** Densitometric analysis of Beclin1 and pULK1 levels relative to β-actin. Protein levels were represented as [%] to β-actin. Data were analyzed by one-way ANOVA with Tukey’s multiple comparison post-test. Data are representative of two independent experiments. *P < 0.05, **P < 0.01, ***P < 0.001 *vs*. controls. n.s; difference not significant.

**Figure 8:**
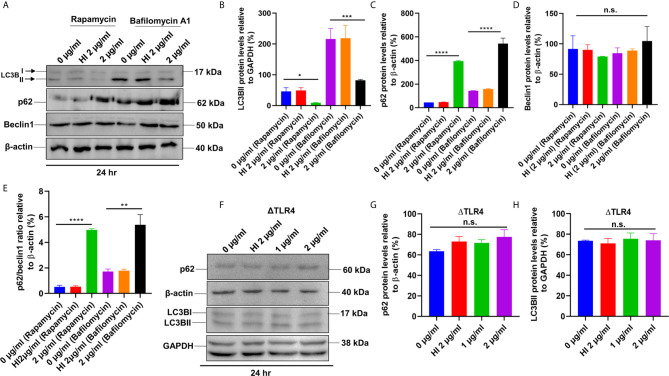
PE6 inhibits autophagy initiation of RAW264.7 cells in the presence of rapamycin and bafilomycin A1. **(A)** Western blot showing the levels of autophagy markers LC3BII, p62, and Beclin1 in RAW264.7 cells treated with PE6 in the presence of rapamycin (200 nM) and bafilomycin A1 (50 nM). Untreated and HI PE6 treated cells were used as negative controls. β-actin and GAPDH were used as loading controls. **(B–E)** Densitometric quantifications of LC3BII, p62, and Beclin1 bands are shown relative to β-actin and GAPDH [%]. p62/Beclin1 ratio is shown as a bar graph. **(F)** Western blot demonstrating the levels of p62 and LC3BII in ΔTLR4 mouse macrophage cells. β-actin and GAPDH were used as loading controls. Untreated and HI PE6 treated cells were used as negative controls. **(F–H)** Densitometric analysis of p62 and LC3BII bands relative to β-actin. Data were analyzed by one-way ANOVA with Tukey’s multiple comparison post-test. Data are representative of two independent experiments. *P < 0.05, **P < 0.01, ***P < 0.001, ****P < 0.001 *vs*. controls. n.s; difference not significant.

**Figure 9:**
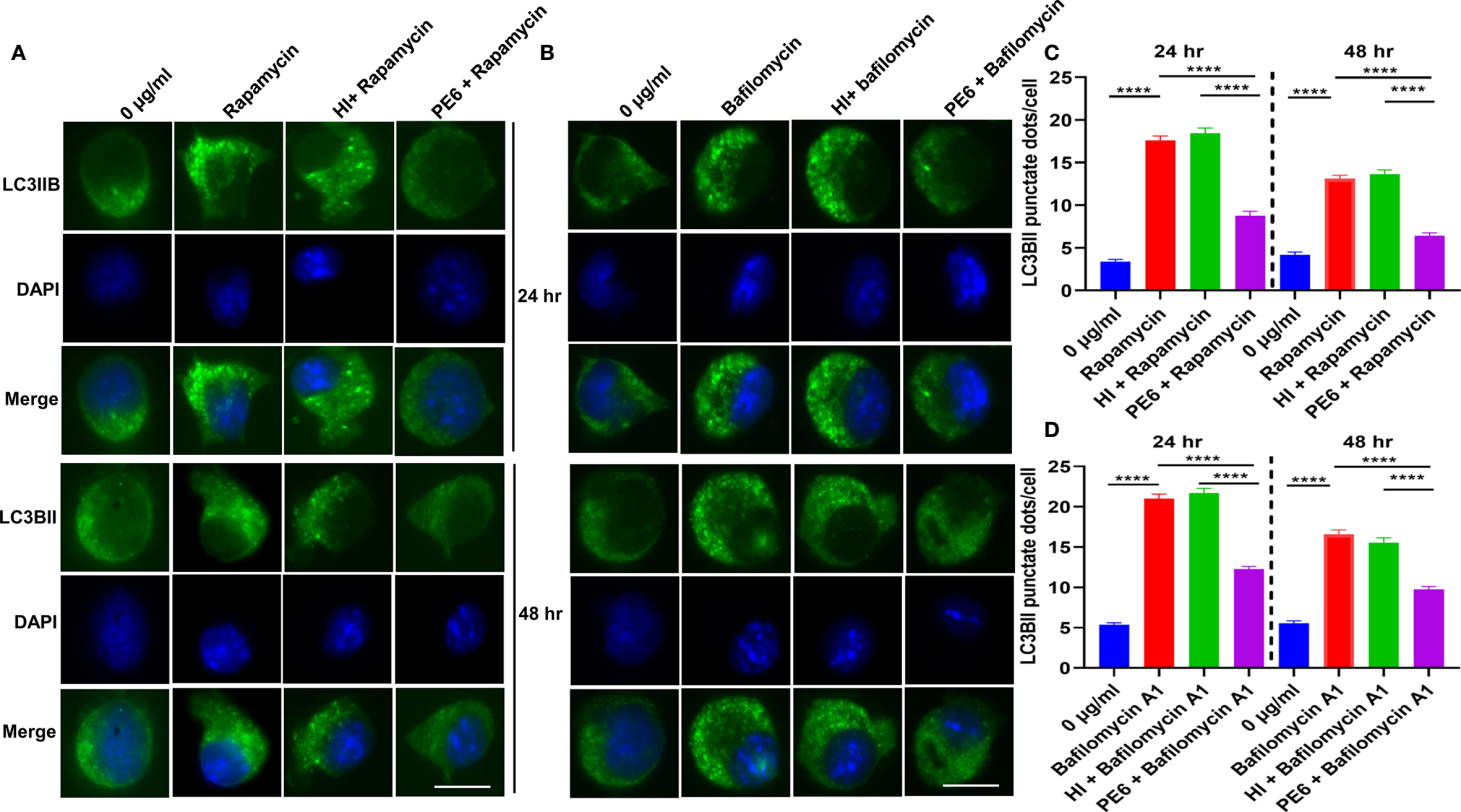
PE6 inhibits the formation of LC3BII punctate foci in treated RAW264.7 macrophage cells in the presence of pharmacological agents rapamycin and bafilomycin A1. **(A)** Analysis of immunofluorescence microscopic images showing the LC3BII foci in untreated, rapamycin (200 nM) treated, HI + rapamycin-treated, and PE6 + rapamycin-treated RAW264.7 cells at 24 and 48 h. DAPI was used to mark the nucleus. Untreated and rapamycin-treated cells were used as negative and positive controls, respectively. **(B)** Immunofluorescence microscopic images demonstrating the LC3BII foci in untreated, HI treated, and PE6 treated RAW264.7 cells in the presence of bafilomycin A1 (50 nM). Untreated and bafilomycin A1 treated cells were used as negative and positive controls. **(C, D)** The average number of LC3BII punctate foci were counted using Axio-vision software and represented as a bar graph. Scale bars indicate 10 µM. Data were analyzed using paired T-test with the Kruskal–Wallis post-test. Data are representative of three independent experiments. ****P < 0.001 *vs*. controls.

**Figure 10:**
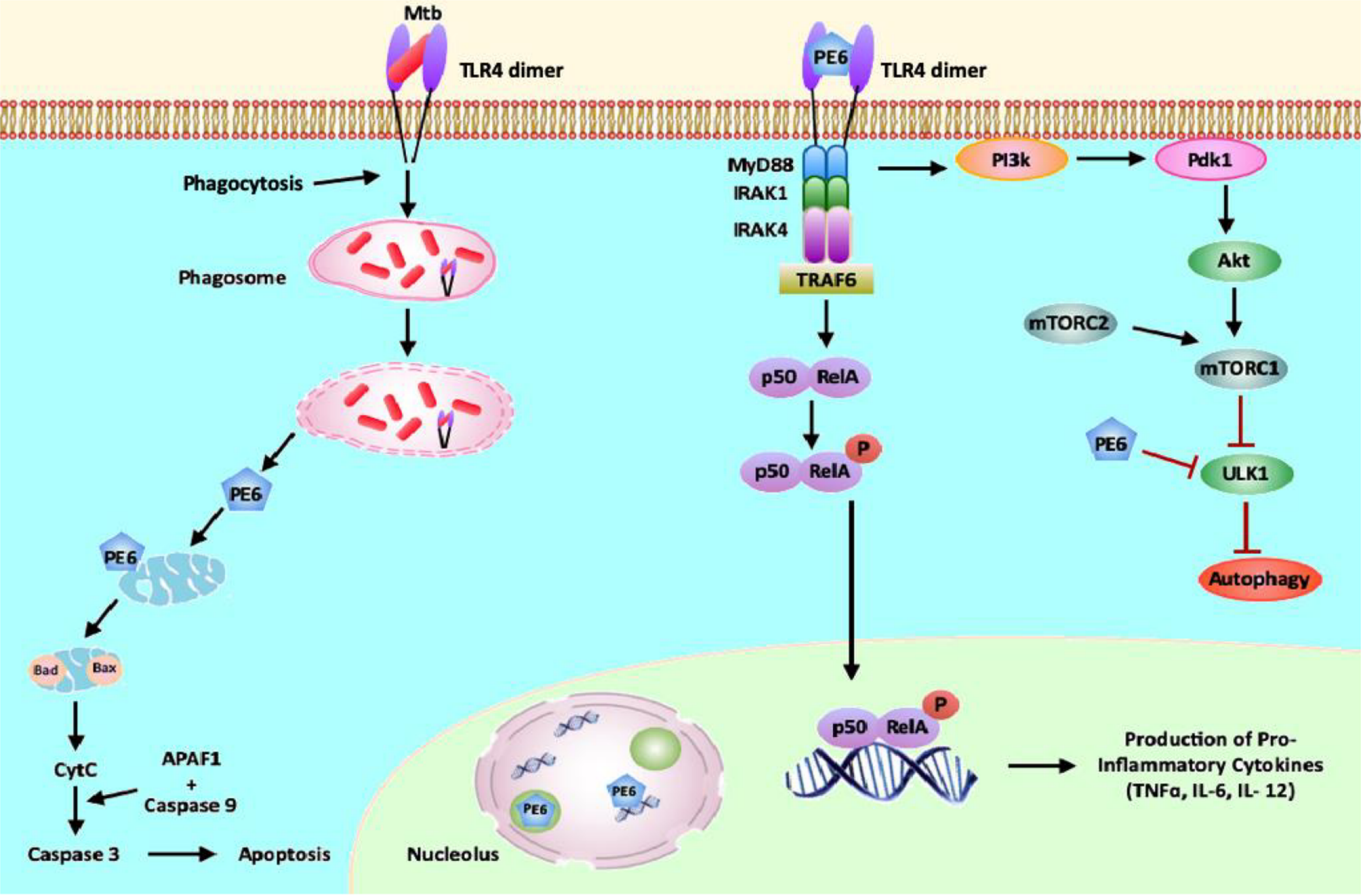
PE6 interacts with surface immune receptor TLR4 and affects downstream signaling cascade. PE6 is a specific TLR4 agonist that recruits adaptor protein Myd88 to affect downstream signaling. Engagement of PE6 to TLR4 activates canonical NFĸB signaling pathways involving NFĸB1 (P50) and RelA (P65). Activation of canonical NFĸB signaling induces increased production of proinflammatory cytokines TNF-α, IL-6, and IL12. PE6 differentially targeted into nucleus/nucleolus and mitochondria, controls the expression of Nucleolin, indicating possible exploitation of Nucleolin functions to modulate host-directed defenses generated against *M. tb*. PE6 induces increased apoptosis through the production of pro-apoptotic molecules Bax, Cytochrome C, and pcMYC. PE6 inhibits autophagy initiation *via* repression of autophagy initiating kinase ULK1 and activation of autophagy master regulator MtorC1 mediated by TLR4. Like other virulence effectors of *M. tb*, PE6 displays moonlighting function to dampen and subdue innate host defenses targeted against the pathogen for successful pathogenesis.

### PE6 Has DNA as Well as Iron-Binding Activity

The modeled PE6 structure displays a predominant α-helical structure as earlier observed with other PE/PPE proteins (42). The unusually high pI (12.1) and its nuclear localization prompted us to look for positively charged amino acid patches and DNA binding capability. DNABind *in-silico* analysis of modeled PE6 structure predicted large positive charge patches around 8–12, 24–27, 114–120, and 159–170, a.a locations, which can function as potential DNA binding sites. Preliminary DNA binding was confirmed using H-Dock, which predicted PE6 DNA binding capability with high probability (**Figure 5A**). To confirm our *in-silico* predictions on nucleic acid binding, we performed fluorometric titrations of PE6 with dsDNA. PE6 exhibited a strong association with dsDNA, apparent from its dissociation constant (1.4 ± 0.15 µM) (**Figures 5B, D**). Further, to study DNA binding mediated stabilization of PE6, we performed FTS assays. FTS assay is based on the principle that small-molecule binding stabilizes the protein through H-bond interactions and entropic contributions. The mid-point of change in fluorescence signal (melting temperature, T_m_) was determined for PE6 alone and dsDNA. Interestingly, during the thermal ramping of PE6 alone, we observed an increase in fluorescence intensity, whereas, in the presence of dsDNA, the characteristic sigmoidal curve was observed with a T_m_ of 56.2 ± 1.1°C (**Figures 5D, E**). The unusual increase in fluorescence intensity of PE6 alone upon thermal ramping could be attributed to protein-compaction or oligomerization, as observed in many intrinsically disordered proteins (43).

PE-PPE proteins have been reported to have a role in iron acquisition and assimilation (44). Functional prediction of PE6 using i-Tasser based coach and cofactor analysis revealed Asn62, Gly92, and His95 as potential iron-binding residues. To validate the *in vitro* iron chelating activity of PE6, we carried out fluorometric titrations of PE6 with Fe^3+^. The apparent dissociation constant was 2.6 ± 0.4nm, indicating an intense iron sequestering activity (**Figures 5C, F**). Similarly, T_m_ was determined for PE6–Fe^3+^ complex, which was observed to be 48.5 ± 0.8°C (**Figures 5C, D**). These findings suggest that PE6 is capable of binding to DNA and iron.

### Recombinant *M. smegmatis* Expressing PE6 Displayed Robust Intracellular Survival Within RAW264.7 Cells, Pronounced Under Low Iron Conditions

We generated recombinant *M. smegmatis* containing *pe6* (**Figures S3A–C**) and analyzed the *in-vitro* growth kinetics and survival inside the macrophage cells using CFU assay. We found that recombinant *M. smegmatis* containing *pe6* showed significantly slower growth kinetics compared to vector alone transformed *M. smegmatis* (**Figure S3E**). Interestingly, *pe6* containing *M. smegmatis* showed higher survival inside macrophages (**Figure S3F**). As iron is an essential micronutrient that is important for the survival of *M. tb*, it usually sequesters iron from the host to maintain its cellular physiology. Various mycobacterial proteins have been implicated in maintaining this steady influx of iron within the bacterium, including PE/PPE family proteins PPE36 and PPE62 (13). After ascertaining that PE6 has an iron-binding site within its sequence, we hypothesized that it could play a role in sequestering iron from the bacterial environment. To confirm this, recombinant *M. smegmatis* containing *pe6* and vector alone and wild-type cells were cultured in the absence and presence of iron chelator BPS and complemented with Fecl3. The *pe6* containing *M. smegmatis* showed robust growth in liquid and solid media in comparison to vector alone transformed cells in the presence of iron chelator BPS (**Figures 5G, H**). We also observed that the growth of *M. smegmatis* containing vector alone was severely hampered when iron was chelated from the media using an iron chelator as opposed to *M. smegmatis* containing *pe6* (**Figures 5G, H**). The growth of *M. smegmatis* transformed with vector alone was more strongly inhibited in iron-depleted solid media, while *M. smegmatis* containing *pe6* displayed robust growth in iron-depleted conditions (**Figures 5G, H**).

Furthermore, we also analyzed the CFU of liquid-grown wild-type, vector-alone transformed cells and *M. smegmatis* containing *pe6* in the presence of iron chelator BPS. Recombinant *M. smegmatis* containing *pe6* showed robust growth compared to vector alone transformed *M. smegmatis* as evidenced by increased CFU (**Figure S3G**). These results suggest that PE6 is involved in acquiring iron from the surrounding media for bacterial survival, as earlier shown for other PE/PPE family proteins PPE36 and PPE62.

### PE6 Induces Caspase-Dependent Apoptosis of RAW264.7 Cells and Activates the ER-Associated UPR Pathway

We initially investigated the cell viability of RAW264.7 cells upon protein treatment using MTT assay. RAW264.7 cells were treated with various concentrations of PE6 for a period of 24 h. We observed that 5 µg/ml of PE protein-induced measurable cell death (**Figure S4A**). We analyzed the cell death using Flow Cytometry and Western blot analysis to confirm its role in apoptosis regulation. Macrophage cells were treated with various concentrations of PE6 protein (5, 7.5, and 10 µg/ml), and apoptosis was assayed using Propidium iodide (PI) and Annexin V staining. FACS analysis revealed that PE6 induced enhanced apoptosis of macrophage cells in a concentration-dependent manner, as evidenced by increased Annexin V staining (**Figures 6A, B**).

We next examined the activation status of initiator and executioner caspases, Caspases 9 and 3, by employing Western blot analysis. Macrophage cells, upon treatment with various concentrations of PE6 for 24 h, display increased cleavage of full-length Caspase 9 and increased generation of cleaved Caspase 3 (**Figures S4B–E**), a hallmark of caspase activation. To further strengthen these observations, macrophage cells were infected with *pe6* containing *M. smegmatis* and vector alone harboring *M. smegmatis* for 24 h and subjected to Western blot analysis. PE6 induced increased production of pro-apoptotic proteins Bax and Cytochrome C and also induced increased activation of pro-apoptotic transcription factor pcMyc, which is involved in apoptosis induction (**Figures 6C–H**).

The unfolded protein response (UPR) pathway plays a critical role in the induction of apoptosis. We accordingly investigated whether PE6-induced apoptosis is through the activation of the ER linked to the UPR pathway. RAW264.7 cells were infected with recombinant *M. smegmatis*, and UPR markers Atf6, Chop, Ire1α, Calnexin, BIP, and Eif2α were analyzed by Western blot analysis. Consistent with our hypothesis, *pe6* containing *M. smegmatis* induced higher UPR markers than the vector alone infected macrophage cells (**Figure 6I**). These observations unravel the fact that PE6 is involved in the induction of apoptosis through pro-apoptotic Bax, Cytochrome C, and activation of transcription factor pcMyc together with Caspases 3 and 9 activation and activation of ER-mediated UPR pathway.

### PE6 Inhibits Cellular Autophagy in RAW264.7 Cells

After unraveling that PE6 induces apoptosis in treated macrophage cells in a dose-dependent manner, we investigated the manipulation of the autophagy pathway by PE6 protein. The cellular autophagy pathway was analyzed by measuring the levels of classical parameters: conversion of LC3BI to LC3BII and the utilization of autophagy substrate p62/SQSTM1 and Beclin1. We treated RAW264.7 cells with different concentrations of PE6 (1 and 2 µg/ml) and analyzed the levels of autophagy markers utilizing Western blot analysis. Cells treated with PE6 protein decreased the conversion of LC3BI to LC3BII and the increased accumulation of autophagy substrate p62 (**Figures 7A–E**). However, we did not observe any change in the Beclin1 protein (**Figures 7A, C**). These results indicated inhibition of autophagy by PE6 protein in a dose-dependent manner. We also calculated the p62/Beclin1 ratio that confirmed the autophagy flux reduction (**Figure 7E**). PE6 also inhibited autophagy activation, initiating kinase ULK1 as revealed by its increased inhibitory phosphorylation (**Figures 7A, F**). Activation of MtorC1 kinase culminates in the inhibition of ULK1, which suppresses autophagy initiation. We further confirmed these observations by infecting recombinant *M. smegmatis* to RAW264.7 cells and analyzed the activatory phosphorylation of MtorC1 and inhibitory phosphorylation of ULK1 using Western blot analysis. Infection with *pe6* containing *M. smegmatis* induced the activatory phosphorylation of MtorC1 (Ser2448) and increased inhibitory phosphorylation of ULK1 (Ser757), whereas no change in the Beclin1 level was observed (**Figures 7G–I** and **Figure S4F**). Ser757 phosphorylation by MtorC1 suppress ULK1 activation and its interaction with AMPK. We further analyzed the levels of LC3BII and autophagy substrate p62 after infection with *M. smegmatis* containing *pe6* and vector alone cells to macrophage cells. Western blot analysis showed that PE6 inhibited the conversion of LCBI to LC3BII and induced the accumulation of autophagy substrate p62 (**Figures S4G–I**). These results confirmed that PE6 reciprocally regulates cell death attributes of the host cell, i.e., apoptosis and autophagy. We went further to investigate the mechanism of autophagy inhibition by PE6 protein. RAW264.7 cells were treated with PE6 and autophagy inducer rapamycin and inhibitor bafilomycin A1, followed by Western blot analysis. Rapamycin is an autophagy inducer that inhibits autophagy master regulator MtorC1, whereas bafilomycin A1 is an autophagy inhibitor that inhibits autophagosome fusion to the lysosome and used to study autophagy flux. We found that PE6 inhibited autophagy even in the presence of rapamycin and bafilomycin A1, which was indicated by the reduced conversion of LC3BI to LC3BII and increased accumulation of p62 autophagy substrate to the PE6 treated cells along with an increased p62/Beclin1 ratio (**Figures 8A–E**). We also studied whether the inhibition of autophagy by PE6 is TLR4 dependent. ΔTLR4 macrophage cells were treated with different concentrations of PE6 protein (1 and 2 µg/ml), followed by Western blot analysis. We did not observe any difference in autophagy substrate levels p62 and LC3BI to LC3BII conversion and the p62/Beclin1 ratio after PE6 treatment (**Figures 8F–H**). To further strengthen our findings, we studied the inhibitory effect of PE6 on cellular autophagy in the presence of rapamycin (200 nm) and bafilomycin A1 (50 nm) in a time-dependent manner; we used immunofluorescence microscopic analysis and analyzed the LC3BII puncta formation in the presence of these pharmacological agents. We observed significantly reduced numbers of LC3BII punctate foci, the indicator of autophagic membrane dynamics, in PE6 treated samples (**Figures 9A, B**). The number of punctate foci was counted in various fields from three independent experiments and shown as bar graphs (**Figures 9C, D**). These results suggest that PE6 efficiently inhibited the cellular dynamics of autophagy initiation in RAW264.7 macrophage cells by repressing the formation of LC3BII associated punctate foci formation. These findings suggest that PE6 inhibits autophagy initiation mediated by MtorC1 activation and ULK1 suppression.

## Discussion

This study demonstrates that recombinant PE6 induces the robust production of proinflammatory cytokines TNF-α, IL-12, and IL-6. Many PE proteins, including PE27 and PE_PGRS11, activate antigen-presenting cells to produce proinflammatory cytokines (45, 46). Apart from innate immune activation, many of the proteins from this family are known to activate adaptive immune responses (47). We show that PE6 is a specific TLR4 agonist as it interacts with cell surface innate immune receptor TLR4 in RAW264.7 macrophage cells, whereas the interaction was absent in mice macrophage cells lacking the TLR4 receptor. We also demonstrate that PE6 induces enhanced expression and organization of TLR4 at the membrane, which is a hallmark of its activation. Our investigation suggests that PE induces proinflammatory cytokines’ secretion upon interacting with immune receptor TLR4 at the cell surface to execute its immunomodulatory functions. It is important to mention that the activities of bacterial virulence factors are usually modular, and the best way to demonstrate this would have been by testing different truncations of the recombinant PE6 in terms of molecular interaction between TLR4, which we have not been able to do.

Increased production of proinflammatory cytokines is a prerequisite for the activation of macrophages and surface activation markers MHC-II, CD80, and CD86, which leads to M1 macrophage polarization (48, 49). Activation of macrophages links innate and adaptive immune responses to mount an effective immune response against the invading pathogen. Macrophages can be activated by soluble and cell-wall associated *M. tb* antigens mediated by distinct TLRs protein on the cell surface (50). Lipomannan of *M. tb* induces macrophage activation *via* TLR2, mediated by the adaptor protein Myd88 and characterized by increased TNF-α and nitric oxide (51). *M. tb* 19-kDa lipoprotein is a TLR2 agonist that induces robust production of proinflammatory cytokines, TNF-α, IL-12, and IL-6 by macrophages and shows powerful immunomodulatory properties (52). Similarly, 38-kDa lipoprotein interacts with innate immune receptor TLR2 and TLR4. It induces enhanced TNF-α, IL-12, and IL-6 (53). TLR4 is one of the pattern recognition receptors that recognize various exogenous and endogenous ligands, including LPS, fibronectin, heat shock proteins, and hyaluronan oligosaccharides (54).

Production of proinflammatory cytokines is majorly governed by the activation of NFĸB and map kinase signaling cascades (MAPK) and downstream signaling emanating from TLRs (55, 56). NFĸB, in turn, can also be activated by proinflammatory cytokines, including TNF-α and IL1β (55). Although TLRs are structurally diverse, they commonly activate similar downstream signaling, including activation of IĸB kinase and NFĸB (57). The canonical NFĸB pathway is activated by microbial antigens as well as proinflammatory cytokines TNF-α and IL1β and generally involves RelA (P65) and P50 (NFĸB1) complexes. PE6 induces activation of the canonical NFĸB pathway and elicits increased expression and activation of P50 and P65 subunits. Our results also demonstrate that PE6 induced secretion of proinflammatory cytokines are dependent on TLR4 and adaptor protein Myd88. Therefore, we hypothesize that PE6 mediated activation of canonical NFĸB activation facilitates the sustained production of proinflammatory cytokines and induces macrophage M1 polarization.

Apoptosis and autophagy of macrophages are two critical innate host defenses that play essential roles in determining the pathogenesis or host defense against *M. tb*. Apoptosis plays an essential role in defense against intracellular pathogens by preventing the release of intracellular pathogens and, thus, the spread of the *M. tb* infection (58, 59). Emerging evidence suggests that alveolar macrophages activate apoptosis after *M. tb* infection as an innate immune defense (60). Apoptosis-induced cell death of *M. tb* infected macrophages is generally associated with mycobacterial killing and efficient induction of T cell responses *via* enhanced antigen presentation (61, 62). It is worth mentioning that avirulent strains of mycobacteria (BCG and H_37_Ra) are potent inducers of apoptosis in macrophages compared to virulent strains such as H_37_Rv (63, 64).

Interestingly, several of the *M. tb* virulence effectors induce ER stress-mediated apoptosis of the host cell, such as 38-kDa antigen, ESAT6, HBHA, PE_PGRS5, and PE_PGRS33 (26, 65–67). On the contrary, many of the *M. tb* effectors are known, which inhibit apoptosis, including NuoG and PknE (68, 69). Virulent *M. tb* inhibits apoptosis at an early stage of infection, whereas when the bacterial burden is high within the infected macrophages, it induces apoptosis for successful dissemination (26). Our results revealed that PE6 is a potent inducer of macrophage apoptosis. It induces apoptosis through the activation of Caspases 3 and 9. The infection of RAW264.7 cells with recombinant *M. smegmatis* containing *pe6* induced increased expression of pro-apoptotic proteins Bax and cytochrome C and enhanced activation of transcription factor pcMyc. Pro-apoptotic Bax forms a pore in the mitochondrial membrane and facilitates the release of Cytochrome C, which activates the homodimerization of APAF-1 and recruitment of Caspase 9, thereby its activation (70).

We also elucidated that PE6 induced increased ER stress markers, transcription factor Atf6, Chop, Calnexin, BIP, Ire1α, and Eif2α. These observations are consistent with the increased expression of pro-apoptotic factors. Therefore, the findings suggest that PE6 induces the UPR pathway-mediated apoptosis of macrophages likely late in the infection cycle after excessive mycobacterial burden to aid in the apoptosis-mediated spread of mycobacterial cargos to other phagocytes in the vicinity.

Autophagy is also a critical innate defense mechanism against invading intracellular pathogens, including *M. tb.* Recent evidence implicates that various bacterial pathogen target autophagy *via* producing specific virulence factors that enable intracellular survival of the pathogens and help in dissemination (71). These two critical innate immune defense pathways, autophagy, and apoptosis are linked both positively and negatively. Extensive cross-talks exist between the processes, and these pathways can be activated sequentially within the same cell by various stresses. Generally, activation of autophagy blocks the induction of apoptosis, and *vice versa*, activation of caspases shut off the autophagic process. Several pieces of evidence suggest that *M. tb* has developed sophisticated immune evasion strategies that interfere with autophagy (19), thereby enhancing its intracellular survival by limiting the presentation of its antigen by MHC-II molecules. Many mycobacterial virulence factors have been implicated in immune evasion through autophagy blockades, such as type VII ESAT6 secretion system, PE_PGRS47, and enhanced intracellular survival protein Eis (20, 21, 23). In the present study, we decipher the inhibitory effect of PE6 on macrophage autophagy.

PE6 inhibits autophagy, as evidenced by the lower conversion of LC3BI to LC3BII and reduced consumption of autophagy substrate p62. Although there is no reduction in the Beclin1 levels, we observed an increased p62/Beclin1 ratio, indicating autophagy inhibition. Furthermore, the observation that PE6 inhibits autophagy in the presence of rapamycin and bafilomycin A1 strengthens our finding and suggests that PE6 hamper autophagy initiation. Using recombinant *M. smegmatis* containing *pe6*, we demonstrate that PE6 inhibits activation of autophagy initiating kinase ULK1 and activates autophagy regulator MtorC1. ULK1, together with Atg13 and RB1CC1, initiates autophagophore biogenesis (72). AMPK and MtorC1 are two critical regulators of ULK1 activation through phosphorylation. The master regulator of autophagy, MtorC1, phosphorylates ULK1 at Ser757, which ultimately culminates in the repression of its activation by preventing its interaction with AMPK. Therefore, our observation suggests that PE6 inhibits autophagy initiation *via* inhibiting ULK1 activity (72).

The reductive evolution in *M. tb* has endowed its proteins to have moonlighting functions (73, 74). Apart from immunomodulation and regulation of cell death pathways, the PE/PPE proteins of *M. tb* are targeted to various host cell organelles to manipulate diverse cellular functions, a glaring example of protein promiscuity with diverse implications in pathogenesis (75, 76). Furthermore, PE_PGRS5 protein localizes to the ER in a PGRS domain-dependent manner, unlike PE_PGRS33. Targeting of PE_PGRS5 to the ER induced UPR mediated host cell apoptosis *via* enhanced production of Atf4 and Chop and disrupted calcium ion homeostasis (26). The PE_PGRS33 protein has also been shown to localize into the mitochondria both upon transient transfection and after infection. Furthermore, upon mitochondrial targeting, the PE_PGRS33 protein-induced host cell apoptosis and necrosis to regulate *M. tb* pathogenesis (40). Additionally, the PPE2 was shown to localize into the nucleus of the host cell (41). Recently, it has been shown that the C-terminal region of PPE37 protein localizes to the nucleus under low iron conditions and induces caspase-dependent apoptosis to favor the dissemination of the bacteria within the host (25).

Our experiment to determine the cellular localization of PE6 in host cells revealed a new observation wherein PE6 exhibited a time-dependent differential localization pattern within transfected HEK293T cells. It localizes to the nucleus/nucleolus as well as mitochondria. The shift in its localization from the nucleus/nucleolus to mitochondria increased at 48 → 72 h. The observed unexpected co-localization of Nucleolin and PE6 in the cytoplasm and its interaction with PE6 strongly indicate possible exploitation of Nucleolin functions by *M. tb.* Strong DNA binding of PE6, its nucleus/nucleolus targeting, and presence of Spt5 C-terminal nonapeptide repeat binding Spt4 sequence suggest that PE6 may be utilized by *M. tb* in order to compromise host cell machinery by manipulating RNA polymerase II and other regulatory complexes, modulating ribosomal biogenesis, apoptosis, and autophagy. As per the best of our information, this is the first report showing an *M. tb* protein that localizes into the host nucleolus, and further studies are warranted to delineate this interesting and unexplored aspect.

Another exciting aspect unraveled in this study was the interaction of PE6 with Fe^3+^. Iron is a vital nutrient for *M. tb* virulence (44), and *M. tb* sequesters iron by secreting siderophores, mycobactin’s, and carboxy-mycobactin’s with a different affinity (77). ESX-3 type VII secretion system has been demonstrated to play an essential role in the acquisition of iron and its homeostasis (78). A recent finding has shown the role of a surface PE/PPE family protein in heme uptake (79). However, the complete understanding of iron acquisition physiology and pathways in *M. tb* remains unknown. Our finding that PE6 increases the growth capability of recombinant *M. smegmatis* in the iron chelator’s presence suggest its possible iron acquisition role. The cell envelops association and secretory nature of PE6 support this observation and indicates PE6 as an essential virulence factor.

Summarizing the observations from this study, we propose a model (**Figure 10**) in which *M. tb* PE6 interacts with the innate immune receptor TLR4. Interaction of PE6 to TLR4 results in activating a proinflammatory signaling cascade by induction of canonical NFĸB signaling.

Additionally, PE6 is targeted differentially to nucleus/nucleolus and mitochondria in a time-dependent manner and regulates the level and unexpected distribution and co-localization in the cytoplasm with the nuclear protein Nucleolin. Mitochondrial localization of PE6 likely activates apoptosis through the induction of pro-apoptotic proteins and can induce the ER stress-dependent UPR pathway. Our results also reveal that PE6 inhibits host innate defense mechanism autophagy *via* suppressing autophagy, initiating kinase ULK1, and activating MtorC1 kinase. These results strongly suggest that the PE6 induced modulation of autophagy and apoptosis represents a key virulence strategy used by *M. tb* through PE6 to replicate and survive within macrophages, and production of proinflammatory cytokine secretion may cause successful pathology for dissemination and virulence. This multipronged attack by a single effector protein of *M. tb* additionally advocates its candidature as a valuable drug target to combat tuberculosis.

## Data Availability Statement

The original contributions presented in the study are included in the article/**Supplementary Material**. Further inquiries can be directed to the corresponding authors.

## Ethics Statement

The Institutional Animal Ethics Committee of the National Institute of Pathology approved the present study (Code no: 1801). New Zealand white female rabbit was maintained at Central Animal Facility of National Institute of Pathology as approved by the Institutional Animal Ethics Committee. After completing experimental procedures, the rabbit was returned to Central Animal Facility, AIIMS, for rehabilitation.

## Author Contributions

Conceptualize the project: NE and SH. Data curation and analysis: NS, MS, NQ, JS, NE, and SH. Funding acquisition and investigation: NE and SH. Writing manuscript draft: NS, MS, NE, and SH. Manuscript review and editing: MS, JAS, NE, and SH. Project administration and supervision: NE and SH. All authors contributed to the article and approved the submitted version.

## Funding

JAS is funded by the Start-up Research Grant from UGC and DST-SERB. SH and NE are supported by DBT North-East Grants BT/PR23099/NER/95/632/2017 and BT/PR23155/NER/95/634/2017 by the Department of Biotechnology, Ministry of Science and Technology (MoS & T), Government of India (GoI).

## Conflict of Interest

The authors declare that the research was conducted in the absence of any commercial or financial relationships that could be construed as a potential conflict of interest.

## Supplementary Material

Supplementary Figure 1 PE6 localizes to multiple cellular fractions of *M. tb*. **(A)** Cellular localization of PE6 in various purified fractions of *M. tb*. 20µg of purified *M. tb* fractions were separated in SDS-PAGE, and Western blotting was performed using the anti-PE6 antibody. PE6 was detected majorly in the cell wall, and culture filtrate and minor presence were detected in the whole cell lysate, cytosol, and total membrane. Localization of GroEL2 and Mpt32 were used as controls.

Supplementary Figure 2 PE6 contains a sequence signature for secretion signal, mitochondria, and nuclear localization. PSORTII **(** https://psort.hgc.jp/cgi-bin/runpsort.pl) and TargetP (http://www.cbs.dtu.dk/cgi-bin/webface2.fcgi?jobid=604DDB3900004BF5C348503C&wait=20) predicted the presence of a mitochondrial localization signal, and LocSigDB (http://genome.unmc.edu/LocSigDB/) shows the presence of a nuclear localization signal. SignalP ver 4.1 (http://www.cbs.dtu.dk/cgi-bin/webface2.fcgi?jobid=604DDC2E00004BF560E4F38B&wait=20) was used to predict the presence of N-terminal signal peptide. SMART domain database (http://smart.embl-heidelberg.de/smart/job_status.pl?jobid=473014622420041615712400xwHNiRMETj) was used to predict Spt5 C-terminal nonapeptide repeat binding Spt4. A schematic representation of PE6 with various sequence signature features is given on the right side of the figure.

Supplementary Figure 3
*pe6* containing *M. smegmatis* exhibit increased survival within macrophage cells. **(A)** SDS-PAGE showing the purified PE6 protein. Purified PE6 and proteinase K digested and heat-inactivated (HI) protein was run in SDS-PAGE and stained with Coomassie brilliant blue. **(B)** Western blot showing the purified PE6 detected using an anti-his antibody. **(C)** *pe6* cloned in pST-ki (*pe6*), and pST-ki plasmids were electroporated in competent *M. smegmatis*. Positive clones were selected and checked for the expression of PE6 using an anti-flag antibody. **(D)** Western blot analysis showing the specificity of an anti-PE6 antibody. Dilution of anti-PE6 antibody is shown in the figure. β-actin was used as a loading control. **(E)** The growth of *M. smegmatis* containing *pe6* and *M. smegmatis* vector control was analyzed by measuring OD_600_ nm at different time points, as shown in the figure. *M. smegmatis* vector control cells showed significantly faster growth than *M. smegmatis* containing *pe6* cells. **(F)** Intracellular survival of recombinant *M. smegmatis* with infected macrophage cells. Macrophage cells were infected with recombinant *M. smegmatis* at MOI of 1:10. After 24 hr and 48 hr, post-infection cells were lysed in.01% SDS and plated in 7H11 agar plates. Growth was observed after the fourth day of plating. Survival within macrophage cells was represented as CFU. **(G)** Survival of wild-type and recombinant *M. smegmatis* containing *pe6* and vector alone cells in the presence of media alone, iron chelator BPS, and BPS+Fecl3. An aliquot of grown bacteria was plated on a 7H11 agar plate, and CFU was analyzed at various growth points. *M. smegmatis* containing *pe6* showed enhanced survival in the presence of BPS as compared to wild-type and vector-alone transformed cells. Data were analyzed by two-way ANOVA with Sidak’s multiple comparison post-test. Data are representative of 2 independent experiments. *P<0.01, **P<0.01, **P<0.01 *vs*. controls.

Supplementary Figure 4 PE6 induces robust activation of Caspase 9 and Caspase 3. **(A)** The bar graph showing the cytotoxicity of PE6 on macrophage cells. Macrophage cells were treated with various concentration of PE6 (0.5, 1, 2.5, 5, and 10µg/ml) for 24 hr. MTT assay was performed to analyze the cytotoxic effect of PE6 on macrophage cells after 24 hr of post-protein treatment. The percent cytotoxic effect was represented as a bar graph. Proteinase K digested, and heat-inactivated (HI) protein was used as a negative control. **(B)** Western blots showing the activation of Caspase 9 after PE6 treatment to macrophage cells. Macrophage cells were treated with different concentrations of PE6, as shown in the figure, for 24 hr. Antibodies used and the molecular weights are indicated in the figure. **(C)** The bar graph represents the densitometric quantitation of full-length Caspase 9 normalizes to β-actin. **(D)** Western blot showing the level of active Caspase 3 after treatment with various concentrations of PE6 for 24 hr, as shown in the figure. Untreated cells and HI PE6 treated cells were used as negative controls. Staurosporine was used as a positive control. **(E)** Full length and cleaved Caspase 3 were shown in Western blot. Macrophage cells were infected with recombinant *M. smegmatis* for 24 hr. The cell lysate was prepared and run in SDS-PAGE, and Western blotted using an anti-Caspase 3 antibody. Untreated and vector alone transformed cells were used as negative controls. β-actin was used as a loading control. **(G)** Western blots showing the levels of LC3BII and p62 at 24 hr post-infection with recombinant *M. smegmatis* containing *pe6* and vector alone harboring cells. **(H, I)** Densitometric quantitation of LC3BII and p62 protein levels normalized to β-actin and represented as bar graphs. One-way ANOVA analyzed data with Tukey’s multiple comparison post-test. Data are representative of 2 independent experiments. *P<0.05 *vs*. controls.
