# Supplementary figures and images for: Mycobacterium tuberculosis Protein PE6 (Rv0335c), a Novel TLR4 Agonist, Evokes an Inflammatory Response and Modulates the Cell Death Pathways in Macrophages to Enhance Intracellular Survival
Source: Front Immunol. 2021 Jul 12;12:696491. doi: 10.3389/fimmu.2021.696491 (PMC8311496; doi:10.3389/fimmu.2021.696491)

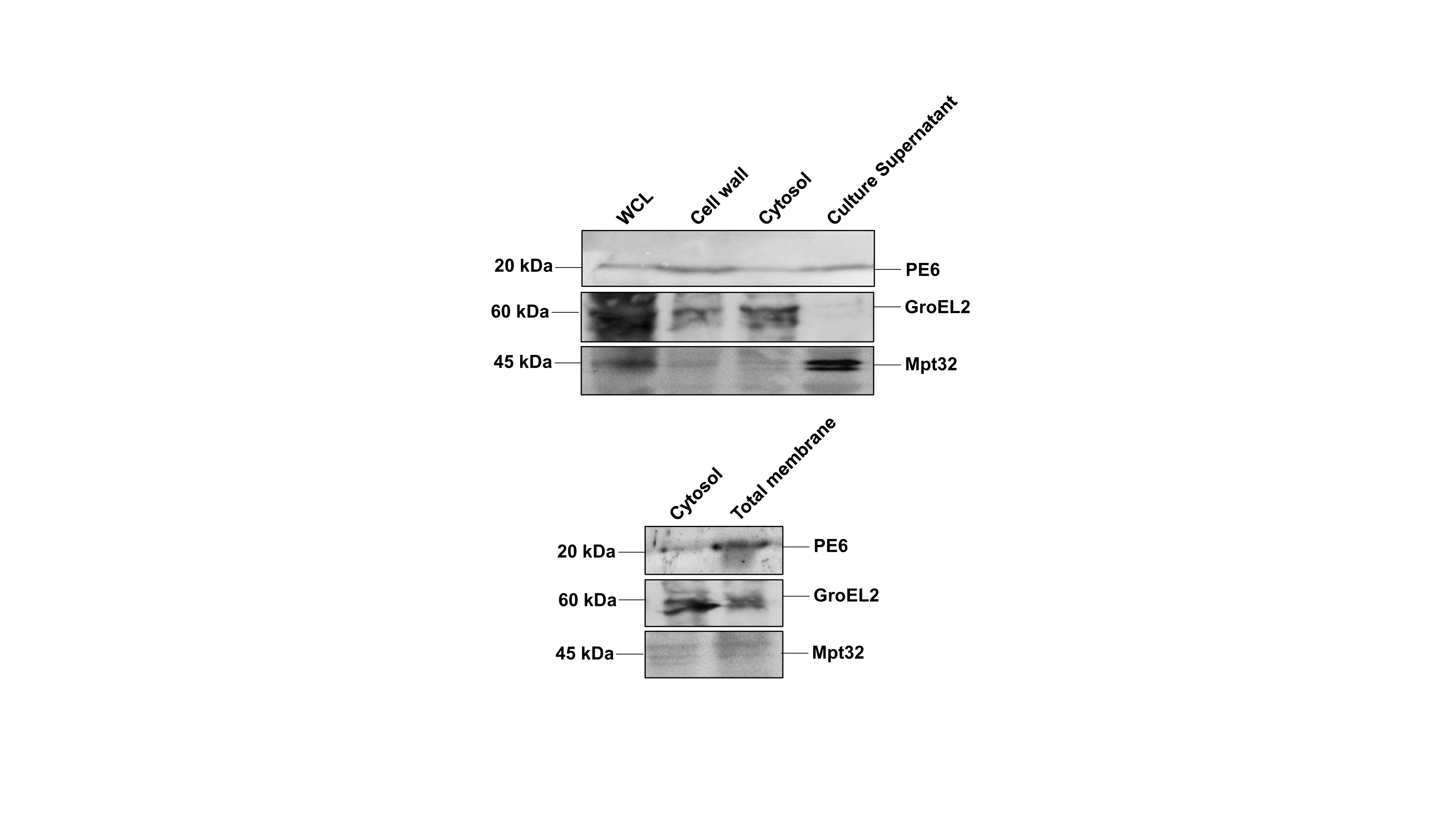

Supplement: Supplementary Figure 1 — PE6 localizes to multiple cellular fractions of M. tb. (A) Cellular localization of PE6 in various purified fractions of M. tb. 20µg of purified M. tb fractions were separated in SDS-PAGE, and Western blotting was performed using the anti-PE6 antibody. PE6 was detected majorly in the cell wall, and culture filtrate and minor presence were detected in the whole cell lysate, cytosol, and total membrane. Localization of GroEL2 and Mpt32 were used as controls. [file Image_1.tif]

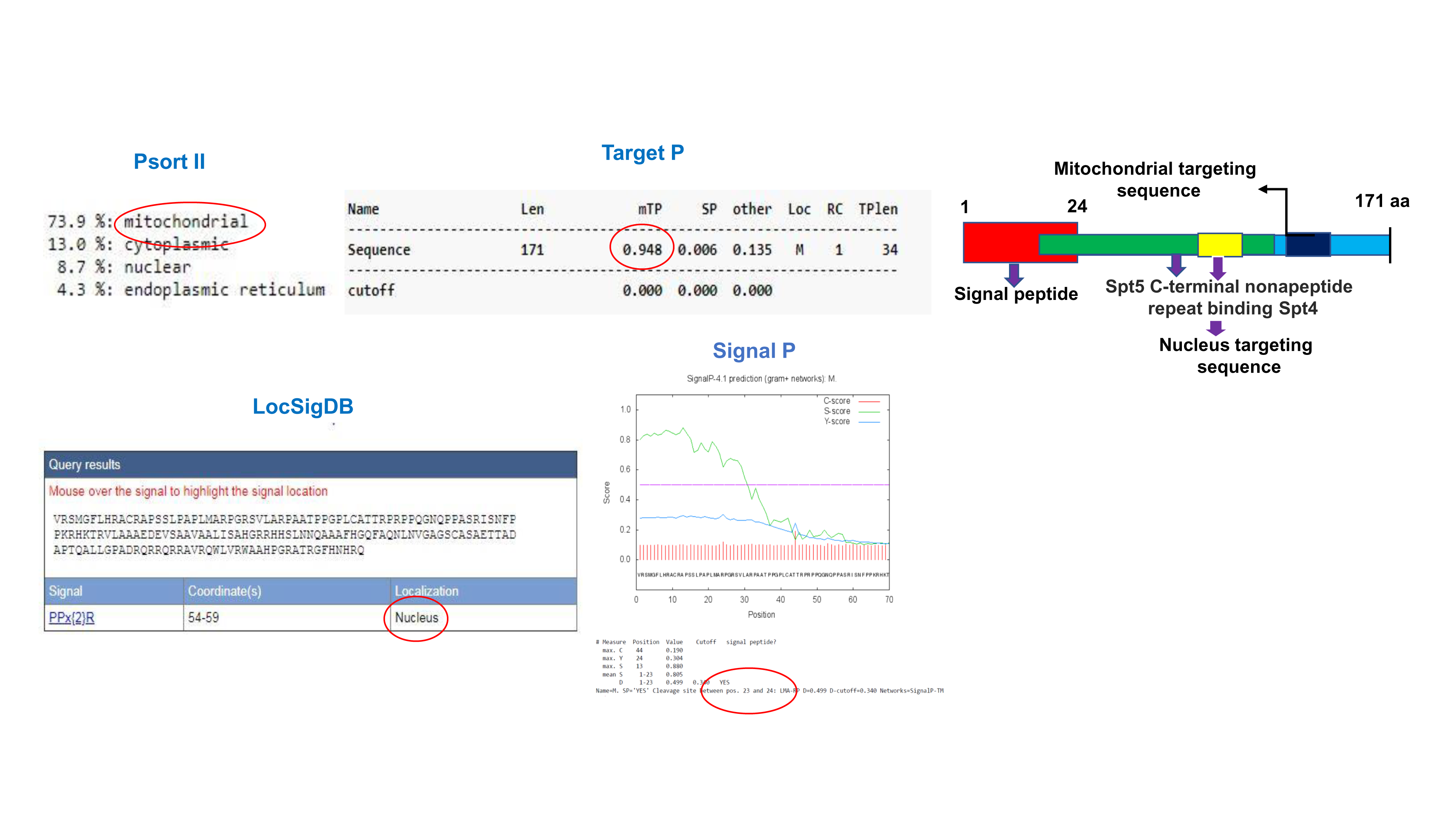

Supplement: Supplementary Figure 2 — PE6 contains a sequence signature for secretion signal, mitochondria, and nuclear localization. PSORTII ( https://psort.hgc.jp/cgi-bin/runpsort.pl) and TargetP (http://www.cbs.dtu.dk/cgi-bin/webface2.fcgi?jobid=604DDB3900004BF5C348503C&wait=20) predicted the presence of a mitochondrial localization signal, and LocSigDB (http://genome.unmc.edu/LocSigDB/) shows the presence of a nuclear localization signal. SignalP ver 4.1 (http://www.cbs.dtu.dk/cgi-bin/webface2.fcgi?jobid=604DDC2E00004BF560E4F38B&wait=20) was used to predict the presence of N-terminal signal peptide. SMART domain database (http://smart.embl-heidelberg.de/smart/job_status.pl?jobid=473014622420041615712400xwHNiRMETj) was used to predict Spt5 C-terminal nonapeptide repeat binding Spt4. A schematic representation of PE6 with various sequence signature features is given on the right side of the figure. [file Image_2.tif]

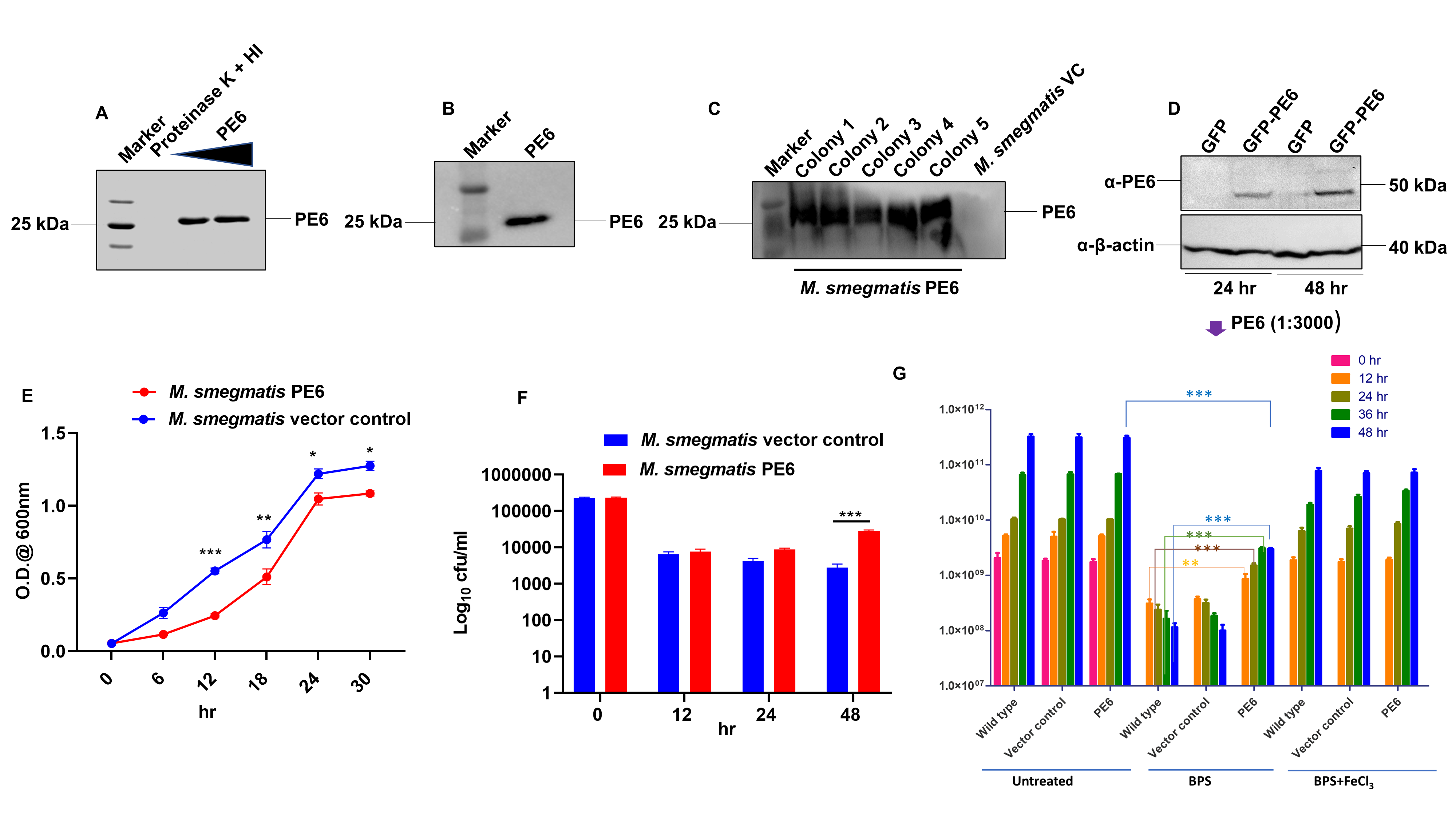

Supplement: Supplementary Figure 3 — pe6 containing M. smegmatis exhibit increased survival within macrophage cells. (A) SDS-PAGE showing the purified PE6 protein. Purified PE6 and proteinase K digested and heat-inactivated (HI) protein was run in SDS-PAGE and stained with Coomassie brilliant blue. (B) Western blot showing the purified PE6 detected using an anti-his antibody. (C) pe6 cloned in pST-ki (pe6), and pST-ki plasmids were electroporated in competent M. smegmatis. Positive clones were selected and checked for the expression of PE6 using an anti-flag antibody. (D) Western blot analysis showing the specificity of an anti-PE6 antibody. Dilution of anti-PE6 antibody is shown in the figure. β-actin was used as a loading control. (E) The growth of M. smegmatis containing pe6 and M. smegmatis vector control was analyzed by measuring OD600 nm at different time points, as shown in the figure. M. smegmatis vector control cells showed significantly faster growth than M. smegmatis containing pe6 cells. (F) Intracellular survival of recombinant M. smegmatis with infected macrophage cells. Macrophage cells were infected with recombinant M. smegmatis at MOI of 1:10. After 24 hr and 48 hr, post-infection cells were lysed in.01% SDS and plated in 7H11 agar plates. Growth was observed after the fourth day of plating. Survival within macrophage cells was represented as CFU. (G) Survival of wild-type and recombinant M. smegmatis containing pe6 and vector alone cells in the presence of media alone, iron chelator BPS, and BPS+Fecl3. An aliquot of grown bacteria was plated on a 7H11 agar plate, and CFU was analyzed at various growth points. M. smegmatis containing pe6 showed enhanced survival in the presence of BPS as compared to wild-type and vector-alone transformed cells. Data were analyzed by two-way ANOVA with Sidak’s multiple comparison post-test. Data are representative of 2 independent experiments. *P<0.01, **P<0.01, **P<0.01 vs. controls. [file Image_3.tif]

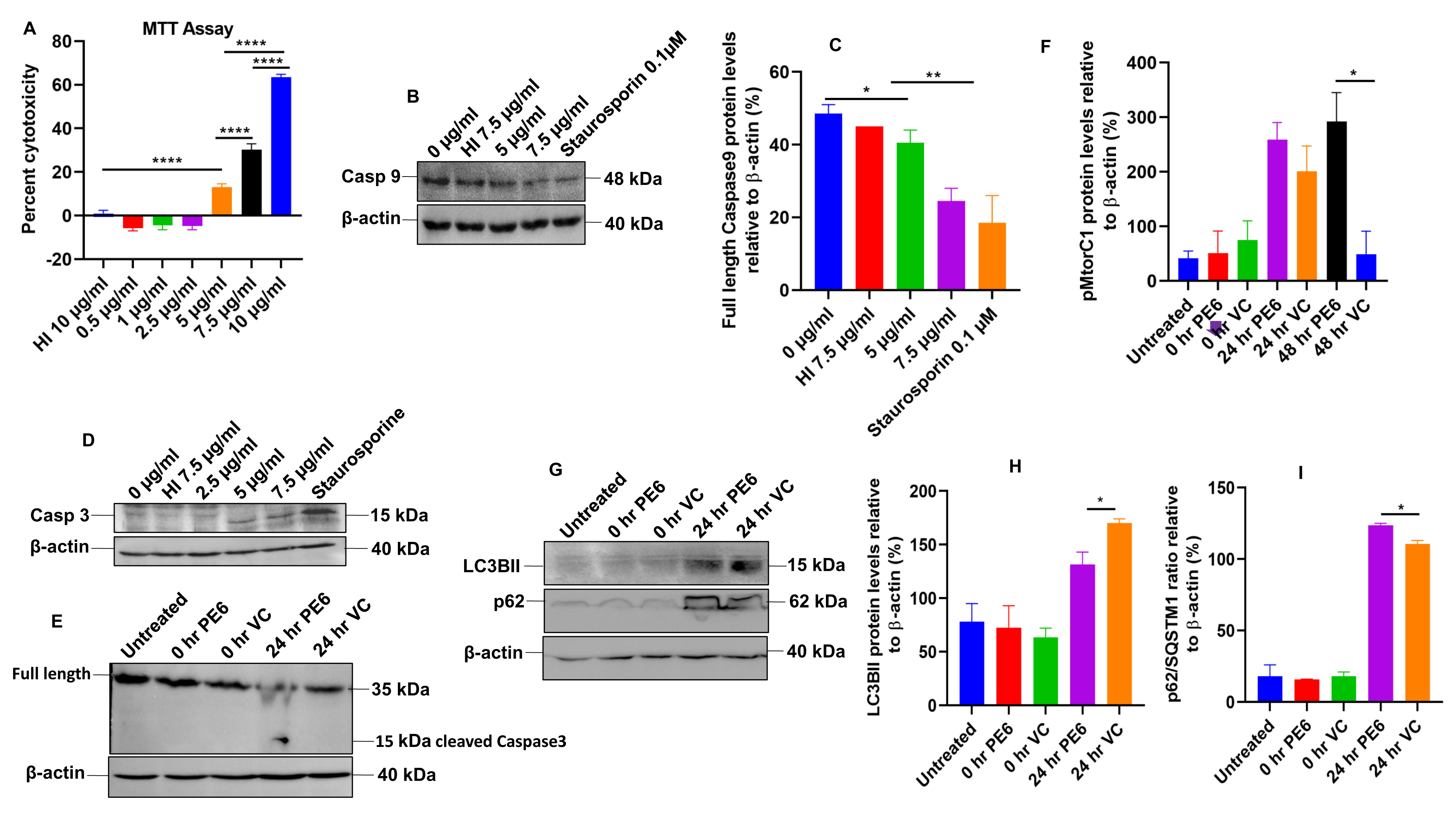

Supplement: Supplementary Figure 4 — PE6 induces robust activation of Caspase 9 and Caspase 3. (A) The bar graph showing the cytotoxicity of PE6 on macrophage cells. Macrophage cells were treated with various concentration of PE6 (0.5, 1, 2.5, 5, and 10µg/ml) for 24 hr. MTT assay was performed to analyze the cytotoxic effect of PE6 on macrophage cells after 24 hr of post-protein treatment. The percent cytotoxic effect was represented as a bar graph. Proteinase K digested, and heat-inactivated (HI) protein was used as a negative control. (B) Western blots showing the activation of Caspase 9 after PE6 treatment to macrophage cells. Macrophage cells were treated with different concentrations of PE6, as shown in the figure, for 24 hr. Antibodies used and the molecular weights are indicated in the figure. (C) The bar graph represents the densitometric quantitation of full-length Caspase 9 normalizes to β-actin. (D) Western blot showing the level of active Caspase 3 after treatment with various concentrations of PE6 for 24 hr, as shown in the figure. Untreated cells and HI PE6 treated cells were used as negative controls. Staurosporine was used as a positive control. (E) Full length and cleaved Caspase 3 were shown in Western blot. Macrophage cells were infected with recombinant M. smegmatis for 24 hr. The cell lysate was prepared and run in SDS-PAGE, and Western blotted using an anti-Caspase 3 antibody. Untreated and vector alone transformed cells were used as negative controls. β-actin was used as a loading control. (G) Western blots showing the levels of LC3BII and p62 at 24 hr post-infection with recombinant M. smegmatis containing pe6 and vector alone harboring cells. (H, I) Densitometric quantitation of LC3BII and p62 protein levels normalized to β-actin and represented as bar graphs. One-way ANOVA analyzed data with Tukey’s multiple comparison post-test. Data are representative of 2 independent experiments. *P<0.05 vs. controls. [file Image_4.tif]
