# Supplementary material for: Mycobacterium tuberculosis Protein PE6 (Rv0335c), a Novel TLR4 Agonist, Evokes an Inflammatory Response and Modulates the Cell Death Pathways in Macrophages to Enhance Intracellular Survival
Source: Front Immunol. 2021 Jul 12;12:696491. doi: 10.3389/fimmu.2021.696491 (PMC8311496; doi:10.3389/fimmu.2021.696491)
Supplement: Supplementary file 5 [file Table_1.docx]

Table S1. Bacterial strains and plasmid used in this study

| Strains and plasmids | Description | Source/Ref |
| --- | --- | --- |
| Strains |  |  |
| *E.coli* |  |  |
| BL21(DE3) ClearColi® | *F– ompT hsdSB (rB- mB-) gal dcm lon λ(DE3 [lacI lacUV5-T7 gene 1 ind1 sam7 nin5]) msbA148 ΔgutQΔkdsD ΔlpxLΔlpxMΔpagPΔlpxPΔeptA* | Lucigen |
| DH5α | *F^-^ [ϕ80dΔlacZM15] Δ(lacZYA-argF)U169 deoR recA1 endA1 hsd R17 glnV44 thi-1 gyrA96 relA1* | Gibco-BRL |
| *Mycobacterium* |  |  |
| *M. smegmatis* mc^2^155 | *ept-1* |  |
| Plasmids |  |  |
| pET28a | Km^r^, His tag protein expression vector | Novagen |
| pST-Ki | Km^r^, His and FLAG tag shuttle vector for mycobacterium for constitutive protein expression | (1) |
| pET28a-PE6 | Km^r^, a fragment containing entire PE6 coding region cloned in pET28a | This study |
| pST-Ki PE6 | Km^r^, a fragment containing entire PE6 coding region cloned into pST-Ki insertion vector | This study |
|  |  |  |

1. **Parikh A, Kumar D, Chawla Y, Kurthkoti K, Khan S, Varshney U, Nandicoori VK.** 2013. Development of a new generation of vectors for gene expression, gene replacement, and protein-protein interaction studies in mycobacteria. Appl Environ Microbiol **79:**1718-1729.
