# Supplementary material for: Mycobacterium tuberculosis Protein PE6 (Rv0335c), a Novel TLR4 Agonist, Evokes an Inflammatory Response and Modulates the Cell Death Pathways in Macrophages to Enhance Intracellular Survival
Source: Front Immunol. 2021 Jul 12;12:696491. doi: 10.3389/fimmu.2021.696491 (PMC8311496; doi:10.3389/fimmu.2021.696491)
Supplement: Supplementary file 6 [file Table_2.docx]

Table S2. Primers used in this study.

| **Primer name** | **Nucleotide sequence (5’ 3’)** | **Comment** |
| --- | --- | --- |
| **Gene cloning** |  |  |
| pET28a-PE6 F | ATGAATTCGTGCGGTCCATGGGGTTC | Cloning of PE6 into pET28a and pST-Ki |
| pET28a-PE6 R | ATAAGCTTTTGTCGGTGATTATGGAACCC |  |
| pEGFPN1-PE6 F | ATAAGCTTATGGTGCGGTCCATGGGGTTCTTG | Cloning of PE6 into pEGFPN1 vector |
| pEGFPN1-PE6 R | ATGGATCCTTTTGTCGGTGATTATGGAACCC |  |

PCR Condition used in this study:

| **Condition** | **pET28a PE6 and pST-Ki PE6** | **pEGFPN1- PE6** |
| --- | --- | --- |
| Initial Denaturation | 98°C, 4 min | 98°C, 4 min |
| Denaturation | 98°C, 30 sec | 98°C, 30 sec |
| Annealing | 55°C, 40 sec | 57°C, 40 sec |
| Extension | 72°C, 30 sec | 72°C, 30 sec |
| Final Extension | 72°C, 5 min | 72°C, 5 min |
| Total number of cycles | 30 cycles | 30 cycles |
