# Supplementary material for: Mycobacterium tuberculosis Protein PE6 (Rv0335c), a Novel TLR4 Agonist, Evokes an Inflammatory Response and Modulates the Cell Death Pathways in Macrophages to Enhance Intracellular Survival
Source: Front Immunol. 2021 Jul 12;12:696491. doi: 10.3389/fimmu.2021.696491 (PMC8311496; doi:10.3389/fimmu.2021.696491)
Supplement: Supplementary file 7 [file Table_3.docx]

Table S3. H-bonds and Salt bridge interactions between TLR4 and PE6 protein.

| **S. No. PE6 Distance (nm) TLR4** |
| --- |
| **H-bond interactions** |
| 1 C:ARG 9[ NH1] 2.62 A:MET 358[ O ] |
| 2 C:ARG 9[ NH2] 2.73 A:ASN 359[ O ] |
| 3 C:ARG 24[ NH1] 2.74 A:ASP 500[ OD2] |
| 4 C:ARG 24[ NH2] 2.82 A:SER 453[ OG ] |
| 5 C:ARG 24[ NH2] 2.75 A:ASP 500[ OD2] |
| 6 C:ASN 53[ ND2] 3.23 A:ASP 548[ OD2] |
| 7 C:ASN 53[ ND2] 3.36 A:SER 550[ OG ] |
| 8 C:ARG 67[ NH1] 2.76 A:ASN 472[ OD1] |
| 9 C:ARG 67[ NH2] 2.69 A:ASN 472[ OD1] |
| 10 C:ARG 146[ NH1] 2.72 A:THR 109[ OG1] |
| 11 C:ARG 146[ NH1] 2.66 A:SER 85[ OG ] |
| 12 C:ARG 154[ NH1] 2.66 A:ASN 57[ OD1] |
| 13 C:ARG 154[ NH2] 2.69 A:ASN 57[ OD1] |
| 14 C:TRP 155[ NE1] 2.99 A:ASP 59[ OD2] |
| 15 C:ARG 161[ NE ] 2.76 A:ASN 105[ OD1] |
| 16 C:ARG 161[ NH2] 2.78 A:ASN 129[ OD1] |
| 17 C:ARG 161[ NH2] 2.72 A:ASN 105[ OD1] |
| 18 C:MET 1[ SD ] 3.39 A:ARG 337[ NH2] |
| 19 C:GLN 54[ OE1] 2.61 A:LYS 503[ NZ ] |
| **Salt bridges** |
| 1 C:ARG 24[ NH1] 2.74 A:ASP 500[ OD2] |
| 2 C:ARG 24[ NH1] 3.39 A:ASP 500[ OD1] |
| 3 C:ARG 24[ NH2] 3.95 A:ASP 451[ OD2] |
| 4 C:ARG 24[ NH2] 2.75 A:ASP 500[ OD2] |
